# A cell-based multiplex immunoassay platform using fluorescent protein-barcoded reporter cell lines

**DOI:** 10.1038/s42003-021-02881-w

**Published:** 2021-11-25

**Authors:** Shengli Song, Miriam Manook, Jean Kwun, Annette M. Jackson, Stuart J. Knechtle, Garnett Kelsoe

**Affiliations:** 1 Department of Immunology, Duke University School of Medicine, Durham, North Carolina USA; 2 Department of Surgery, Duke University School of Medicine, Durham, North Carolina USA

**Keywords:** Antibodies, Flow cytometry, Antibody therapy, Assay systems, High-throughput screening

## Abstract

Multiplex immunoassays with acellular antigens are well-established based on solid-phase platforms such as the Luminex^®^ technology. Cell barcoding by amine-reactive fluorescent dyes enables analogous cell-based multiplex assays, but requires multiple labeling reactions and quality checks prior to every assay. Here we describe generation of stable, fluorescent protein-barcoded reporter cell lines suitable for multiplex screening of antibody to membrane proteins. The utility of this cell-based system, with the potential of a 256-plex cell panel, is demonstrated by flow cytometry deconvolution of barcoded cell panels expressing influenza A hemagglutinin trimers, or native human CCR2 or CCR5 multi-span proteins and their epitope-defining mutants. This platform will prove useful for characterizing immunity and discovering antibodies to membrane-associated proteins.

## Abstract (web summary)

Song et al. describe an immunoassay method, which is a multiplex, fluorescence-barcoded cell lines system that enables high-throughput screening of antibodies binding to large number of protein variants in a single-tube manner. They demonstrate the utility of this tool by assessing binding of different antibodies to a panel of influenza hemagglutinin antigens and to epitope/domain map CCR2 and CCR5 antibodies.

## Introduction

Membrane proteins are often critical antigens in humoral responses to infection and physiologically significant in health and disease. They are, therefore, valuable targets in vaccine design and therapeutic antibody development^1^. For many multi-subunit or multi-pass transmembrane proteins, the native conformation is preserved only when the protein is properly expressed on the cell membrane^2^. For this reason, cell-based binding assays using protein antigens expressed on the cell surface are widely used in antibody screening and characterization^3^. A major limitation in these assays is that they are single analyte, or singleplex methods, and consequently time-consuming, sample-depleting, and costly when used to screen samples with high numbers (>10) of antigens. Although multiplex immunoassays based on the Luminex^®^ technology are well-established, only acellular antigens can be immobilized on detection beads with this platform.

Fluorescent cell barcoding (FCB) was developed as a cell-based multiplexing technique^4–7^. Distinct cell populations are labeled with unique signatures of amine-reactive fluorescent dyes of different emission wavelengths and intensities before pooling for exposure to potentially reactive antibody followed by analysis by flow cytometry. The FCB technique improves the throughput of flow cytometry experiments and minimizes staining variability and antibody consumption. Although this FCB approach offers flexibility in barcoding cell samples from different sources, especially primary cells, barcoding, labeling, and quality assurance procedures are required prior to every experiment. In contrast, a panel of reporter cell lines stably barcoded by endogenous expression of fluorescent proteins (FPs) yet capable of expressing select membrane-associated antigens by transduction, would establish a stable and highly reproducible immunoassay platform. Once established, endogenously barcoded reporter cell lines would greatly simplify the FCB approach.

Here we describe a cell-based, multiplex immunoassay platform using reporter cell lines with stable barcoding by FPs. Briefly, a basal cell line was engineered to express different combinations of FPs that can be reliably detected and distinguished in different channels on flow cytometers. This resulted in a panel of FP-barcoded reporter cell lines analogous to barcoded Luminex^®^ beads. Individual reporter cell sub-lines, each bearing a unique combination of FPs, were subsequently engineered to express different cell surface proteins to permit their pooling in a cell-based multiplex immunoassay. Flow cytometry was then used to de-multiplex signals from pooled reporter cell lines by serial gating to identify FP combinations. In this proof-of-concept study, we established a 16-plex basic panel and tested the feasibility of expanding to a 256-plex panel of reporter cell lines. We successfully applied this platform to multiplex detection of antibody binding to cell panels expressing various influenza A hemagglutinin (HA) trimers or human CCR2b and CCR5, and several domain-swap or point mutants that define specific protein domains/epitopes.

## Results

### Generation of a basal reporter cell line suitable for immunoassays

The human chronic myelogenous leukemia cell line K562 was selected as a parental cell line; as a suspension cell line, detachment or digestion treatment is unnecessary, removing the possibility of denaturation of expressed surface antigens. K562 cells also exhibit a high efficiency of transfection and transduction, and are resilient to apoptosis^8^, simplifying the processes of genetic modification and assay processing. A drawback of K562 as a reporter cell line is its expression of the Fcγ receptor, CD32A, which binds most human IgG subclasses with sufficient affinity to raise background thresholds (Supplementary Fig. 1). We therefore knocked out *CD32A* by CRISPR-Cas9-mediated gene targeting (Supplementary Fig. 1). Using genomic DNA sequencing to ensure disabling all *CD32A* alleles, we generated a cloned, basal cell line, K530. Compared with the parental K562 cells, K530 cells are negative for all Fcγ receptors CD16, CD32, and CD64, and show minimal nonspecific binding by human IgG1 (Supplementary Fig. 1).

### Stable barcoding of reporter cell lines with unique combinations of FPs

To introduce stable barcodes into the reporter cells for multiplex detection, we conceived a strategy to use combinations of FPs expressed in the cytosol of reporter cells. The growing toolbox of FPs^9^ coupled with widely used multicolor flow cytometry allows for marking and detecting multiple FPs simultaneously. The number of unique FP combinations is 2^*n*^ where *n* is the number of different FPs. This exponential result provides the “power” for multiplexity. After careful selection and experimental tests, we selected eight FPs, including EBFP2^10^, mTurquoise2^11^, mNeonGreen^12^, mCardinal^13^, mKate2^14^, miRFP703^15^, LSSmOrange^16^, and hmKeima8.5^17^ (Supplementary Table 1). These FPs are characterized by the following: (1) bright fluorescence for good separation and limited spill-over into other fluorescence channels; (2) good photostability and low cytotoxicity; and (3) monomeric FPs to avoid potential Förster resonance energy transfer events between heterologous FPs. We designed and generated a four-color basic panel (Supplementary Figs. 2–5) capable of 16 distinct FP combinations. Extended panels with two (Supplementary Figs. 2 and 3) or four additional colors (Supplementary Figs. 4 and 5) could expand the multiplexity to 64- or 256-plex, respectively. Alternatively, increased multiplexity could be achieved by introduction of a reference membrane protein (e.g., CD8a) or by high/low intensity versions of the same FPs (Supplementary Figs. 6 and 7).

**Fig. 1:**
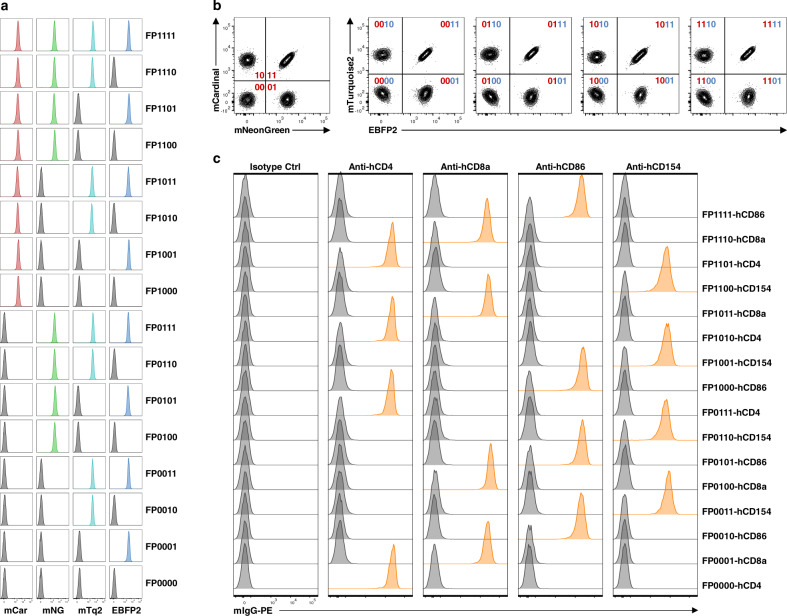
A multiplex immunoassay based on FP-barcoded reporter cell lines. **a** A basic panel of FP-barcoded reporter cell lines. K530 cells were transduced with different combinations of 4 FPs to produce 16 uniquely FP-barcoded reporter cell lines. The absence/presence of fluorescence from FPs EBFP2, mTurquoise2 (mTq2), mNeonGreen (mNG), and mCardinal (mCar) are designated as four digits of binary barcodes as shown on the right of histograms for each individual cell line. **b** Demultiplexing of pooled FP-barcoded reporter cell lines by flow cytometry. **c** The 16 barcoded reporter cell lines were transduced to express human CD4, CD8a, CD86, and CD154 molecules in a shifted pattern relative to FP expression. These cells were pooled and stained with corresponding mouse monoclonal antibodies (as indicated on the top of each histogram) followed by a PE-conjugated anti-mouse IgG antibody. Signals from individual reporter cell lines were demultiplexed as shown in **b** and the binding by corresponding antibodies were plotted as half-offset histograms. In all cases, the detected expression patterns were consistent with antigen expression by barcoded cells before multiplexing as shown on the right of histograms for each individual cell line. Isotype Ctrl, mouse IgG1, κ-isotype control antibody. Data from one experiment are shown. Data from another independent repeat experiment are shown in Supplementary Fig. 8.

We validated the four-color basic panel in supporting 16-plex detection of cell surface molecules. K530 cells were engineered to express all 16 combinations of four FPs from the basic panel, resulting in 16 uniquely FP-barcoded reporter cell lines (Fig. 1a and Supplementary Fig. 8). Pooled cell lines can be demultiplexed by flow cytometry based on patterns of FP expression (Fig. 1b and Supplementary Fig. 8). The growth rates of these 16 FP-barcoded reporter cell lines were determined individually and after pooling; similar growth rates were observed in both conditions (Supplementary Fig. 9). Although proliferation rates of all barcoded cells are similar, variation in growth rates is sufficient such that expansion of pooled cell lines should be limited to three to four doublings or about 3 days of culture to preserve comparable and adequate numbers for the reliable detection of each sub-population. To validate the deconvolution of multiplexed cell populations, we generated 16 cell lines expressing human CD4, CD8a, CD86, or CD154 so that each protein was associated with four unique FP patterns. The pooled cells were stained in single tubes with monoclonal antibody for one of the four human antigens, followed by a common phycoerythrin (PE)-conjugated secondary antibody. Deconvolution by flow cytometry showed high resolution of bound and unbound cells and patterns of binding consistent with antigen expression by barcoded cells before multiplexing (Fig. 1c and Supplementary Figs. 8 and 10).

### Cell surface expression of influenza HA antigens and multiplex detection of binding by reference antibodies

**Fig. 2:**
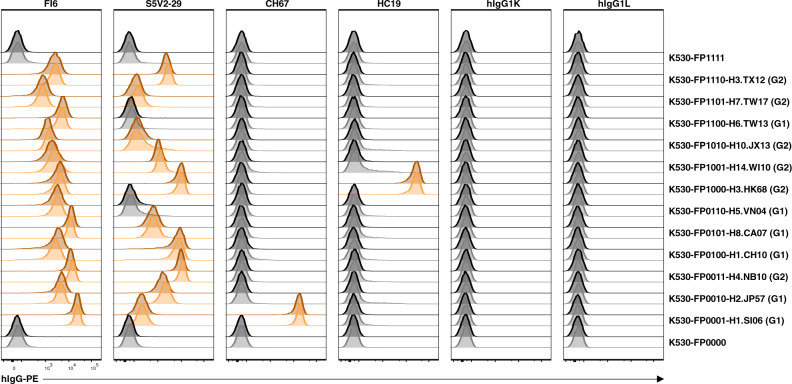
Cell surface expression of influenza A HA antigens and multiplex detection of binding by reference antibodies. FP-barcoded reporter cell lines were transduced to express a panel of influenza A HA antigens of diverse subtypes. Multiplex binding assay was carried out with pooled cell lines. Reference antibodies of human IgG1 isotype were used at 6 µg/ml. hIgG1K and hIgG1L, human IgG1 isotype controls with κ- and λ-light chains, respectively. The binding to each reporter cell line was demultiplexed as shown in Fig. 1. Histograms with MFI values above twofold of the average MFI of internal control cell lines (K530-FP0000 and K530-FP1111) were scored as positive and highlighted in orange. Data from two independent experiments were overlaid, with one experiment shown in light gray or light orange and the other in dark gray or dark orange. H1.SI06, A/Solomon Islands/3/2006(H1N1); H2.JP57, A/Japan/305/1957(H2N2); H4.NB10, A/American black duck/New Brunswick/00464/2010(H4N6); H1.CH10, A/Christchurch/16/2010(H1N1); H8.CA07, A/northern shoveler/California/HKWF1204/2007(H8N4); H5.VN04, A/Viet Nam/1203/2004(H5N1); H3.HK68, A/Hong Kong/JY2/1968(H3N2); H14.WI10, A/mallard/Wisconsin/10OS3941/2010(H14N6); H10.JX13, A/Jiangxi/IPB13/2013(H10N8); H6.TW13, A/Taiwan/2/2013(H6N1); H7.TW17, A/Taiwan/1/2017(H7N9); H3.TX12, A/Texas/50/2012(H3N2). G1, G2, Group 1 and Group 2 HAs, respectively. MFI values for individual histograms are listed in Supplementary Table 2.

Influenza HAs are diverse trimeric membrane proteins. Recombinant HA protein preparation can be challenging as some HA ectodomains are unstable and cell surface expression can be a surer option for obtaining native structure when working with unknown or novel influenza strains. Multiplex immunoassays with collections of cell surface-expressed HA antigens may be useful in influenza vaccine design and immunological monitoring. To demonstrate, we prepared a panel of 12 reporter cell lines expressing the HA trimers of diverse influenza A subtypes and successfully validated their structural integrity with several reference antibodies (Fig. 2 and Supplementary Table 2). The pan-influenza A stem-binding antibody FI6^18^ bound to all the HA antigens tested. The S5V2-29 antibody specific for a conserved interface epitope^19^ bound to a majority of group 1 and group 2 HAs tested. CH67, a neutralizing antibody for H1N1 influenza^20^, bound HA from A/Solomon Islands/3/2006(H1N1) but not A/Christchurch/16/2010(H1N1), an A/California/07/2009(H1N1)-like strain. Antibody HC19, specific for HA A/Hong Kong/JY2/1968(H3N2)^21^, bound to that HA but not to others. This assay may be an alternative to Luminex bead assays in high-throughput screening of antigen-specific and cross-reactive antibodies secreted in clonal cultures of human B cells from influenza-vaccinated subjects^22^.

### Application of the multiplex immunoassay in domain/epitope mapping of antibodies specific for human CCR2 and CCR5

G protein-coupled receptors (GPCRs) are key to many biological functions and are an important class of drug targets, constituting some 34% of US Food and Drug Administration approvals^23^. For these seven-pass transmembrane proteins, mammalian cell surface expression is the most reliable way to preserve native conformation. Domain/epitope mapping of GPCR-specific antibodies can be helpful for structure–function studies of the receptors and for the screening antibodies for therapeutic, diagnostic, and research applications^24^. A cell-based multiplex immunoassay that could accommodate the large number of protein variants needed for domain/epitope mapping in a single-tube could be useful.

**Fig. 3:**
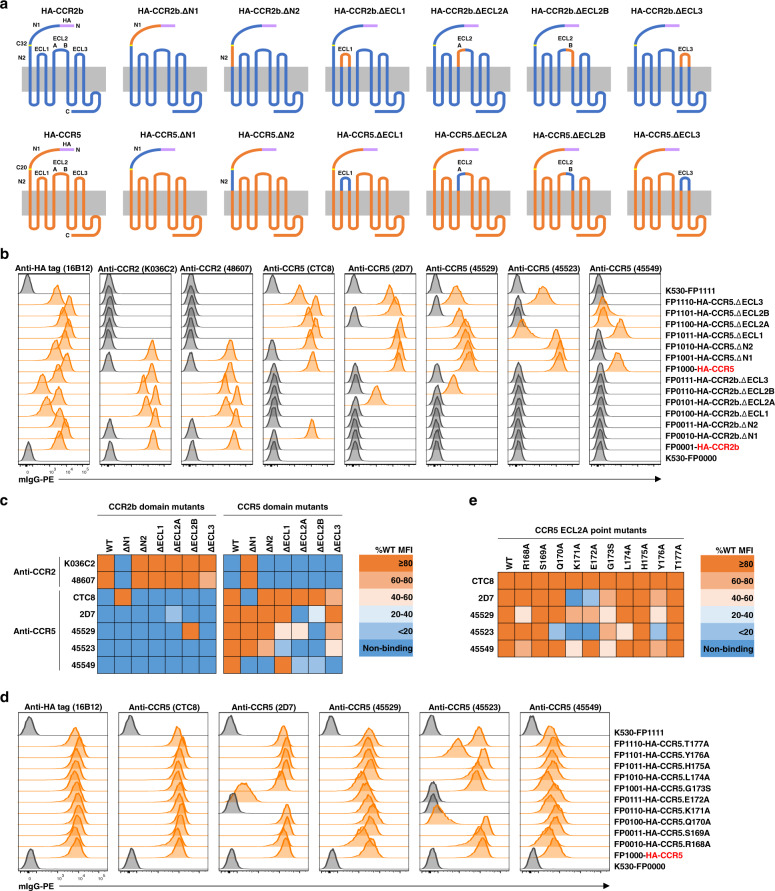
Application of the multiplex immunoassay in domain/epitope mapping of antibodies specific for human CCR2 and CCR5. **a** Schematic diagrams showing CCR2b and CCR5 domain-swapped mutants expressed on reporter cell lines. An N-terminal HA-tag was included to normalize surface expression levels of different mutants. **b** Domain mapping with multiplexed reporter cell lines expressing CCR2b and CCR5 domain-swapped mutants. The binding to each reporter cell line was demultiplexed as shown in Fig. 1. Histograms with MFI values above twofold of background (the average MFI value of internal control cell lines K530-FP0000 and K530-FP1111) were scored as positive and highlighted in orange. **c** Heat map of the relative binding activities of antibodies tested in **b**. Background-subtracted MFI values were first normalized to that with anti-HA-tag antibody and then to corresponding cell lines expressing WT molecules. Percentages of WT MFI values were color-coded according to the key. Negative readouts in **b** were indicated as non-binding. **d** Epitope mapping with multiplexed reporter cell lines expressing CCR5 ECL2A point mutants. The data were analyzed in the same way as in **b**. **e** Heat map of the relative binding activities of antibodies tested in **d** and plotted in the same way as in **c**. All monoclonal mouse antibodies were used at 5 µg/ml. Data from one experiment are shown. Data from another two independent repeat experiments are shown in Supplementary Fig. 11. MFI values for individual histograms are listed in Supplementary Tables 3 and 4.

As an example, a panel of reporter cell lines was prepared, expressing human CCR2b or CCR5 and 12 domain-swapped chimeric mutants (Fig. 3a). Two CCR2-specific and five CCR5-specific antibodies were tested for their binding patterns to these domain-swapped mutants in a multiplex immunoassay (Fig. 3b, Supplementary Fig. 11, and Supplementary Table 3). We found the two uncharacterized CCR2 antibodies to be specific for the CCR2 N1 domain (Fig. 3b, c). Of the previously characterized CCR5 antibodies, CTC8, 2D7, and 45529 bound to the N1, ECL2A, and ECL2B domains, respectively, whereas binding of 45523 and 45549 involved multiple domains (Fig. 3b, c). These observations matched prior reports^25, 26^. The combination of gain- and loss-of-binding readouts across all the ectodomains allowed a semi-quantitative determination of the contribution of each individual ectodomain (Fig. 3c) and revealed more minor-contributing domains unappreciated previously^25, 26^.

Epitope mapping with another panel of reporter cell lines expressing CCR5 mutants with alanine scanning (or glycine-to-serine mutation) in ECL2A domain (Fig. 3d, e, Supplementary Fig. 11, and Supplementary Table 4) showed that, consistent with previous studies^25, 26^, K171 and E172 were critically involved in the binding by 2D7 and 45523. With this scanning panel, another five residues were also determined to be engaged in antibody binding, which were not covered or appreciated in previous studies. The only discrepancy with previous studies^25^ is the role of K171 and E172 in the binding by 45549 and awaits further confirmation. In practice, a full scanning panel can be established to cover every residue in all ectodomains. This multiplex immunoassay can enable high-throughput screening of antibodies with desired specificity and potential function at single-residue resolution in a single-tube manner to expedite therapeutic or diagnostic antibody discovery.

### Potential for expanding the 16-plex reporter cell line panel

**Fig. 4:**
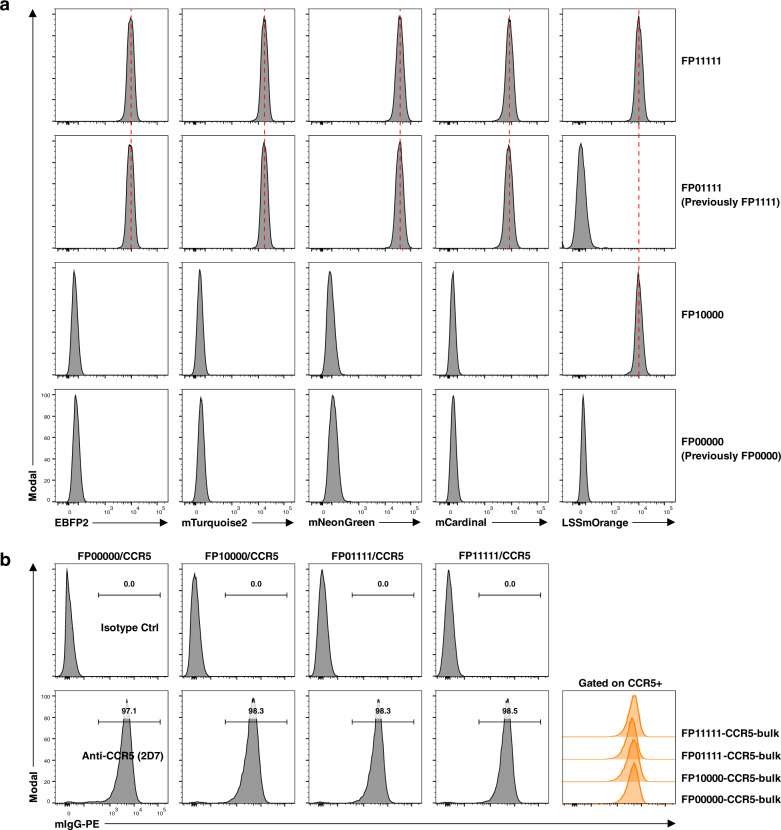
Potential for expanding the 16-plex reporter cell line panel. **a** Generation of reporter cell lines expressing a fifth FP, LSSmOrange. Reporter cell lines expressing none (FP0000) or four (FP1111) FPs in the basic panel were transduced to express LSSmOrange and monoclonal cell lines were generated. The parental cell lines FP0000 and FP1111 were designated as FP00000 and FP01111, respectively, and the derivative cell lines expressing LSSmOrange as FP10000 and FP11111, respectively. Dash lines in red indicate similar express levels of FPs between different cell lines. **b** The vast majority of puromycin-resistant transductants expressed surface CCR5 and the expression levels were comparable among different reporter cell lines with or without a fifth FP. The four reporter cell lines generated above were transduced to express human CCR5. After puromycin selection, the bulk transductants were detected for cell surface express of CCR5. CCR5+ populations were gated and the expression levels were compared in overlaid histogram plot on the right.

To expand the established 16-plex reporter cell line panel, additional transductions for more FPs are necessary. Potentially, the accumulations of genetic insertions in the reporter cell genome could impact the expression and processing of preexisting FPs, other transduced proteins (antigens), and impair cell metabolism or proliferation. To address this concern, we generated a reporter expressing a fifth FP, LSSmOrange. Reporter cell lines FP0000 and FP1111, with none or four preexisting FPs, were pooled and transduced to express LSSmOrange. Similar transduction efficiencies and LSSmOrange intensities were achieved in both parental lines (Supplementary Fig. 12). Monoclonal reporter cell lines were generated from these bulk transductants, with FP10000 expressing only LSSmOrange and FP11111 expressing all five FPs (Fig. 4a). The fluorescent intensities of LSSmOrange in FP10000 and FP11111 were similar; importantly, the fluorescent intensities of the preexisting four FPs remained the same in cell line FP11111 as compared with the parental line FP01111 (Fig. 4a). Similar growth rates were also detected for these four cell lines (Supplementary Fig. 12), within the same range for the 16-plex reporter cell lines (Supplementary Fig. 9).

To confirm that the cell line expressing five FPs (FP11111) remained capable of the expression, folding, and processing of analyte proteins, a complex membrane protein, human CCR5, was tested as an example analyte. Similar transduction efficiencies and surface CCR5 expression levels were detected based on the four cell lines established above (Supplementary Fig. 12). After antibiotic selection, the vast majority (>97%) of successful transductants expressed surface CCR5 at similar levels across all the four cell lines tested (Fig. 4b). These results showed that simultaneous expression of five FPs in the reporter cells did not detectably impair surface expression of a complex membrane protein. We conclude that the 16-plex reporter cell line panel can be readily expanded to 32-plex by introduction of LSSmOrange, and that further expansion by the introduction of additional FPs is likely achievable.

## Discussion

We describe and present here a cell-based multiplex immunoassay platform. A basal cell line was stably barcoded with unique combinations of four endogenous FPs to generate a stable and adaptable panel of 16 distinct reporter cell lines. Analyte protein antigens were then expressed on these reporter cell lines and detected in a multiplex binding assay by flow cytometry. We validated its feasibility in multiplexing and reliability in demultiplexing by flow cytometry. We demonstrated its broad application in multiplex detection of antibody binding to influenza HA and domain/epitope mapping against human CCR2b/CCR5 mutant antigens.

A transient, cell-based multiplex fluorescent barcoding technique, FCB, based on the use of amine-reactive fluorescent dyes was reported previously^4–7^. Although this method enables barcoding of primary cell samples for multiplex detection in single-tube assays, the labeling procedure of each cell reporter must be carried out shortly before every experiment. Obviously, cells labeled in this way cannot be allowed to proliferate as doing so dilutes the label. In contrast, the multiplex platform we have developed uses cell lines with stable FP barcodes to establish membrane antigen-expressing reporter cell line panels for multiplex immunoassays. This platform is especially suitable for the generation of biological or experimental variant libraries based on a same parental cell line background. The downside of the latter platform is that it’s applicable only to cell lines and the initial preparation of reporter cell line panels is a time-consuming process. Once the reporter cell line panel is made, it becomes an unlimited ready-to-use reagent.

In practice, there are several considerations which affect the potential utility of our approach. First, the selection of a proper founder cell line is the principal determinant for generating reporter cell panels that provide both ease of use and robust binding data. Although in theory, any cell line can be used, a suspension cell line with high transfection/transduction efficiency is optimal. In the event that the founder cell line expresses analyte antigen endogenously, an isogenic antigen-negative control cell line must be generated. Second, the stability of introduced transgenes is crucial for the maintenance of reporter cell lines and their reproducibility in immunoassays. In our experience, lentiviral vectors with genetic elements known to prevent epigenetic silencing^27–29^ result in stable and uniform expression of transgenes in the absence of selection pressure. Third, monoclonal reporter cell lines with similar growth rates should be selected to ease challenges in the maintenance of panels with large numbers of reporter cell lines. Reporter cell lines with similar growth rates can be expanded individually and pooled as a single-tube panel and frozen down as single-use stocks. A short-term expansion of the pool after thawing preserves sufficient portions and allows reliable detection for each sub-population (Supplementary Fig. 9). In the maintenance of panels of larger number of reporter cell lines, the portion of each individual cell line for pooling can be adjusted prospectively so that to uniformize the differences after expansion.

In summary, we have developed a cell-based multiplex immunoassay platform that is analogous to the well-established Luminex^®^ platform but for the expression of membrane-associated, not acellular, antigens. The cell-based property of this platform offers flexibility in multiplexing, superiority in supporting surface display of complex membrane proteins in their native conformation, and economy in production once the requisite cell lines are established. These unique features make this platform a valuable tool in humoral response monitoring and antibody characterization. Pooled reporter cell line panels are amenable to high-throughput screening with flow cytometry and in concert can facilitate existing antibody isolation techniques such as hybridoma, single-cell PCR, or single B-cell cultures to aid in antibody discovery and engineering.

## Methods

### Mice and human subjects

Female C57BL/6 mice were obtained from the Jackson Laboratory and maintained under specific pathogen-free conditions at the Duke University Animal Care Facility. Splenocytes were isolated from one 12-week-old mice for RNA extraction. All experiments involving animals were approved by the Duke University Institutional Animal Care and Use Committee (IACUC A128-20-06).

Peripheral blood mononuclear cells (PBMCs) were obtained from one human (*Homo sapiens sapiens*) subject under Duke Institutional Review Board Committee guidelines (IRB Pro00062495). Written informed consent was obtained.

### Original plasmids and DNA templates for gene cloning

pU6-(BbsI)_CBh-Cas9-T2A-BFP (Addgene plasmid # 64323) was a gift from Ralf Kuehn^30^. pLV-EF1a-IRES-Puro (Addgene plasmid # 85132) was a gift from Tobias Meyer^31^. pLB (Addgene plasmid # 11619) was a gift from Stephan Kissler^27^. pMD2.G (Addgene plasmid # 12259) and psPAX2 (Addgene plasmid # 12260) were gifts from Didier Trono. mScarlet-I-mTurquoise2 (Addgene plasmid # 98839) was a gift from Dorus Gadella^32^. pLenti-smURFP-T2A-mCardinal (Addgene plasmid # 80348) was a gift from Erik Rodriguez and Roger Tsien^33^. pMito-miRFP703 (Addgene plasmid # 80000) was a gift from Vladislav Verkhusha^15^. pLSSmOrange-mKate2 (Addgene plasmid # 99868) was a gift from Marc Tramier^34^. Plasmid mNeonGreen-C1 was provided by Allele Biotechnology and Pharmaceuticals, Inc., under a license for non-commercial use.

DNA template coding FP hmKeima8.5 was synthesized (Genscript) based on published amino acid sequences^17^. Coding sequences for other FPs were cloned from plasmids mentioned above, with EBFP2^10^ from pU6-(BbsI)_CBh-Cas9-T2A-BFP, mTurquoise2^11^ from mScarlet-I-mTurquoise2, mNeonGreen^12^ from mNeonGreen-C1, mCardinal^13^ from pLenti-smURFP-T2A-mCardinal, mKate2^14^ and LSSmOrange^16^ from pLSSmOrange-mKate2, and miRFP703^15^ from pMito-miRFP703. Coding sequences for cytoplasmic domain truncated mouse CD4 (amino acid (aa) 1–423), CD8a (aa 1–222), and CD86 (aa 1–268) were cloned from splenocyte cDNA samples from one C57BL/6 mouse. Coding sequences for full-length human CCR2b and CCR5, and cytoplasmic domain truncated human CD4 (aa 1–424), CD8a (aa 1–209), CD86 (aa 1–276), and CD154 (aa 1–240) were cloned from PBMC cDNA samples from one healthy donor.

DNA templates coding influenza HA antigens A/Christchurch/16/2010(H1N1) (GISAID Accession EPI280344) and A/Texas/50/2012(H3N2) (GenBank Accession KJ942744) were synthesized (Gene Universal, Inc.) based on amino acid sequences in the databases. Plasmid containing coding sequences for influenza HA A/Solomon Islands/3/2006(H1N1) (GenBank EU100724, with R540L correction) was provided by Aaron G. Schmidt (Ragon Institute). Plasmids containing coding sequences for other influenza HA antigens used were provided by Stephen C. Harrison (Harvard Medical School), including HA antigens A/Japan/305/1957(H2N2) (GenBank CY014976), A/Hong Kong/JY2/1968(H3N2) (GenBank CY147438), A/American black duck/New Brunswick/00464/2010(H4N6) (GenBank CY138045), A/Viet Nam/1203/2004(H5N1) (GenBank AY818135), A/Taiwan/2/2013(H6N1) (GISAID EPI459855), A/Taiwan/1/2017(H7N9) (GISAID EPI917065), A/northern shoveler/California/HKWF1204/2007(H8N4) (GenBank CY039588), A/Jiangxi/IPB13/2013(H10N8) (GenBank KJ406543), and A/mallard/Wisconsin/10OS3941/2010(H14N6) (GenBank CY133266).

### Plasmid modification and gene cloning

Standard molecular cloning procedures were followed for plasmid modification and gene cloning. Endotoxin-free plasmids were prepared (E.Z.N.A.^®^ Endo-free Plasmid DNA Mini Kit II, Omega Bio-tek) for mammalian cell transfection. Recombinant sequences in all plasmids used were verified by DNA Sanger sequencing (The Duke University DNA Analysis Facility).

The empty lentiviral transfer vector plasmids pLB-EF1a, pLB-EFS, and pLB-EF1a-IRES-Puro (pLB-EXIP; IRES stands for internal ribosome entry site) were constructed by replacing the U6-loxP-CMV-EGFP-loxP cassettes in plasmid pLB with EF1a promoter, EFS core promoter (nucleotide 1-226 of EF1a promoter, with attenuated transcription activity), or EF1a-IRES-Puro cassettes from plasmid pLV-EF1a-IRES-Puro, respectively. pLB-EXIP was further modified to generate bicistronic vectors pLB-EF1a-IRES-EBFP2, pLB-EF1a-IRES-mTurquoise2, pLB-EF1a-IRES-mCardinal, pLB-EF1a-IRES-mCD4, pLB-EF1a-IRES-mCD8a, and pLB-EF1a-IRES-mCD86 by replacing puromycin-resistant gene in pLB-EXIP with coding sequences for corresponding genes following IRES element. pLB-EFS-IRES-mNeonGreen was generated from pLB-EXIP by replacing EF1a promoter with EFS core protomer and puromycin-resistant gene with mNeonGreen coding sequences.

Lentiviral transfer vector plasmids expressing EBFP2, mTurquoise2, mCardinal, mKate2, miRFP703, LSSmOrange, and hmKeima8.5 were generated by cloning of corresponding coding sequences into pLB-EF1a. Lentiviral transfer vector plasmid expressing mNeonGreen was generated by cloning of mNeonGreen coding sequences into pLB-EFS. Human CD4, CD8a, CD86, and CD154 coding sequences (with cytoplasmic domain truncations, see above) were cloned into pLB-EXIP for puromycin-mediated selection. Human CD8a coding sequences were cloned into pLB-EF1a-IRES-mTurquoise2 and pLB-EF1a-IRES-mCardinal, and human CD86 coding sequences were cloned into pLB-EF1a-IRES-EBFP2 and pLB-EFS-IRES-mNeonGreen. These bicistronic expression vectors can co-express human CD8a/CD86 and FPs, with FPs at a lower expression level driven by IRES element. Coding sequences for influenza HA antigens, human CCR2b, CCR5, and their domain-swapped or point mutants were cloned into pLB-EXIP.

### Culture, transfection, and transduction of mammalian cell lines

HEK 293T and K562 cell lines were purchased from ATCC. HEK 293T cells were cultured in Dulbecco’s modified Eagle’s medium (Gibco) supplemented with 10% heat-inactivated HyClone fetal bovine serum (FBS) (Cytiva), 10 mM HEPES buffer, and 55 µM 2-Mercaptoethanol (all Gibco). K562 and derivative cell lines were maintained in RPMI-1640 medium (Gibco) supplemented with 10% heat-inactivated HyClone FBS, 10 mM HEPES buffer, 1 mM sodium pyruvate, 1× MEM NEAA, 55 µM 2-Mercaptoethanol, 100 units/ml penicillin, and 100 µg/ml streptomycin (all Gibco). For all K562-derivative cell lines, monoclonal cell lines were established by single-cell sorting (see below) and used in binding assays in this study.

Single-guide RNAs (sgRNAs) targeting human *CD32A* exon 1 were designed with the online tool (http://crispr.mit.edu). sgRNAs used in this study were sgRNA-hCD32A-1 (5′-AGCAGCAGCAAAACTGTCAA-3′), sgRNA-hCD32A-2 (5′-ATGTATGTCCCAGAAACCTG-3′), and a negative control sgRNA (5′-TGTCATGCGTCACTTAGTGC-3′). Corresponding DNA oligos were synthesized and cloned into plasmid pU6-(BbsI)_CBh-Cas9-T2A-BFP. K562 cells were transfected with CRISPR-Cas9 targeting plasmids using Lipofectamine 3000 Transfection Reagent (Invitrogen). Ninety-six hours after transfection, the cells were collected for flow cytometry analysis and single-cell sorting (see below).

Lentiviral transfer vector plasmids were co-transfected into HEK 293T cells with packaging plasmids pMD2.G and psPAX2 using Lipofectamine 3000 Transfection Reagent (Invitrogen). Forty-eight hours after transfection, culture supernatants were collected and filtered through 0.45 µm polyvinylidene difluoride membrane filters (Millipore). K562-derivative cell lines were transduced with the filtered supernatants containing lentiviral vectors by spinoculation at 1000 × *g* for 45 min at 32 °C. For transductions with pLB-EXIP-based vectors, cells were selected with puromycin (Sigma, 2 µg/ml) between 3 and 7 days after transduction. Seven days after transduction, the cells were collected for flow cytometry analysis and single-cell sorting (see below).

### Antibodies

Monoclonal antibodies used in this study included the following: FITC-conjugated anti-human CD32A (hCD32A-FITC, clone IV.3, STEMCELL 60012FI), hCD32-APC (clone FLI8.26, BD 559769), hCD16-PE (clone 3G8, BioLegend 302007), hCD32-PE (clone FLI8.26, BD 550586), hCD64-PE (clone 10.1, BioLegend 305007), hCD4 (clone SK3, BioLegend 344602), hCD8a (clone HIT8a, BioLegend 300902), hCD86 (clone BU63, BioLegend 374202), hCD154 (clone 24-31, BioLegend 310802), mCD8a-PerCP-eFluor710 (mouse CD8a, clone 53-6.7, ThermoFisher 46-0081), mCD86-PE-Vio770 (clone PO3.3, Miltenyi Biotec 130-105-135), mCD4-APC-Fire750 (clone RM4-5, BioLegend 100568), hCD58-PE (clone MEM-63, ThermoFisher MA1-10256), HA-tag (clone 16B12, BioLegend 901533), hCCR2 (clone K036C2, BioLegend 357201), hCCR2 (clone 48607, R&D Systems MAB150), hCCR5 (clone 2D7, BD 555991), hCCR5 (clone CTC8, R&D Systems MAB1801), hCCR5 (clone 45523, R&D Systems MAB181), hCCR5 (clone 45529, R&D Systems MAB184), and hCCR5 (clone 45549, R&D Systems MAB183). Isotype control antibodies included the following: Mouse IgG1 κ-isotype control (Rockland 010-001-330), Human IgG1κ (hIgG1K, Southern Biotech 0151K-01), and Human IgG1 Lambda (hIgG1L, Southern Biotech 0151L-01). Secondary antibodies included the following: Goat Anti-Human IgG-PE (Southern Biotech 2040-09), Goat Anti-Mouse IgG, and Human ads-PE (Southern Biotech 1030-09). Influenza HA-specific antibodies FI6^18^, S5V2-29^19^, CH67^20^, and HC19^21^ were prepared as recombinant human IgG1 antibodies as described^35^.

### Cell surface staining, flow cytometry analysis, and single-cell sorting and cloning

Cultures of K562 and derivative cells were collected, centrifuged at 300 × *g* for 2 min at 4 °C, and resuspended in staining buffer (phosphate-buffered saline supplemented with 2% heat-inactivated FBS). After incubation with antibodies at 4 °C in the dark for 30 min, cells were washed with staining buffer and resuspended in staining buffer for either secondary staining following the same procedure above or stored on ice for flow cytometry analysis or single-cell sorting. For cell counting, CountBright™ Absolute Counting Beads (Invitrogen) were added into cell suspensions prior to flow cytometry analysis.

Flow cytometry analysis was carried out using either BD FACSCanto II cytometer (Duke Cancer Institute Flow Cytometry Shared Resource) or BD LSR II cytometer (The Duke Human Vaccine Institute (DHVI) Research Flow Cytometry Facility). Single-cell sorting was performed with BD Aria II (The DHVI Research Flow Cytometry Facility). The bulk cell line after transfection or transduction were stained with corresponding antibodies and single cells expressing FP or antigen of interest were sorted into 96-well flat-bottom plates containing 100 µl/well of the complete RPMI medium above supplemented with 20% heat-inactivated FBS. Nine days after sorting, healthily proliferating cell clones were transferred into 24-well plates for further expansion. Three days later, individual monoclonal cell lines were validated for the expression of FP or antigen of interest and a single clone with a uniform expression level was selected for further engineering or used as a reporter cell line in immunoassays.

### Data analysis

FlowJO (Version 10.7.2), Chromas (Version 2.6.6) and Microsoft Office (Version 1808) were used to analyze data and prepare figures for publication.

### Statistics and reproducibility

The reproducibility of flow cytometry detections of the expression of FPs and cell surface molecules was confirmed by two or three independently repeated experiments as indicated in figure legends and shown as Supplementary Figures. The proliferation rates of reporter cell lines were determined with two or three independent cultures for each time point as indicated in corresponding figure legends.

### Reporting summary

Further information on research design is available in the Nature Research Reporting Summary linked to this article.

## Supplementary information

Supplementary Information

Reporting Summary

## Data Availability

All raw data generated during this project are available from the authors upon request. Licensing from Allele Biotechnology and Pharmaceuticals, Inc., is needed prior to material transfer of plasmids containing mNeonGreen sequences. All other plasmids are available from the authors upon request.
