# Supplementary material for: A cell-based multiplex immunoassay platform using fluorescent protein-barcoded reporter cell lines
Source: Commun Biol. 2021 Nov 25;4:1338. doi: 10.1038/s42003-021-02881-w (PMC8617053; doi:10.1038/s42003-021-02881-w)
Supplement: Supplementary file 1 — Supplementary Information [file 42003_2021_2881_MOESM1_ESM.pdf]

A Cell-Based Multiplex Immunoassay Platform using Fluorescent Protein-Barcoded Reporter Cell Lines

Shengli Song[1], Miriam Manook[2], Jean Kwun[2], Annette M. Jackson[1,2], Stuart J. Knechtle[2] and Garnett Kelsoe[1,2,*]

[1] Department of Immunology, Duke University School of Medicine, Durham, North Carolina, USA

[2] Department of Surgery, Duke University School of Medicine, Durham, North Carolina, USA

* Corresponding author, email: garnett.kelsoe@duke.edu

Supplementary Materials

Supplementary Figure 1	The generation of K530 cell line
Supplementary Figure 2	FPs selected for multicolor labeling and detection on BD FACSCanto II flow cytometer
Supplementary Figure 3	Data from an independent repeat experiment for Supplementary Figure 2
Supplementary Figure 4	FPs selected for multicolor labeling and detection on BD LSR II flow cytometer
Supplementary Figure 5	Data from an independent repeat experiment for Supplementary Figure 4
Supplementary Figure 6	Alternative strategies to increase multiplicity of labeling and detection on BD FACSCanto II flow cytometer
Supplementary Figure 7	Data from an independent repeat experiment for Supplementary Figure 6
Supplementary Figure 8	Data from an independent repeat experiment for Figure 1
Supplementary Figure 9	Growth rates of the 16 FP-barcoded reporter cell lines
Supplementary Figure 10	Gating strategy for demultiplexing of pooled FP-barcoded reporter cell lines
Supplementary Figure 11	Data from two independent repeat experiments for Figure 3
Supplementary Figure 12	Expanding the 16-plex reporter cell line panel by introducing a fifth FP
Supplementary Table 1	FPs selected for multi-color barcoding of reporter cell lines
Supplementary Table 2	MFI values of individual histograms shown in Figure 2
Supplementary Table 3	MFI values of individual histograms shown in Figures 3b, S11a and S11c
Supplementary Table 4	MFI values of individual histograms shown in Figures 3d, S11b and S11d

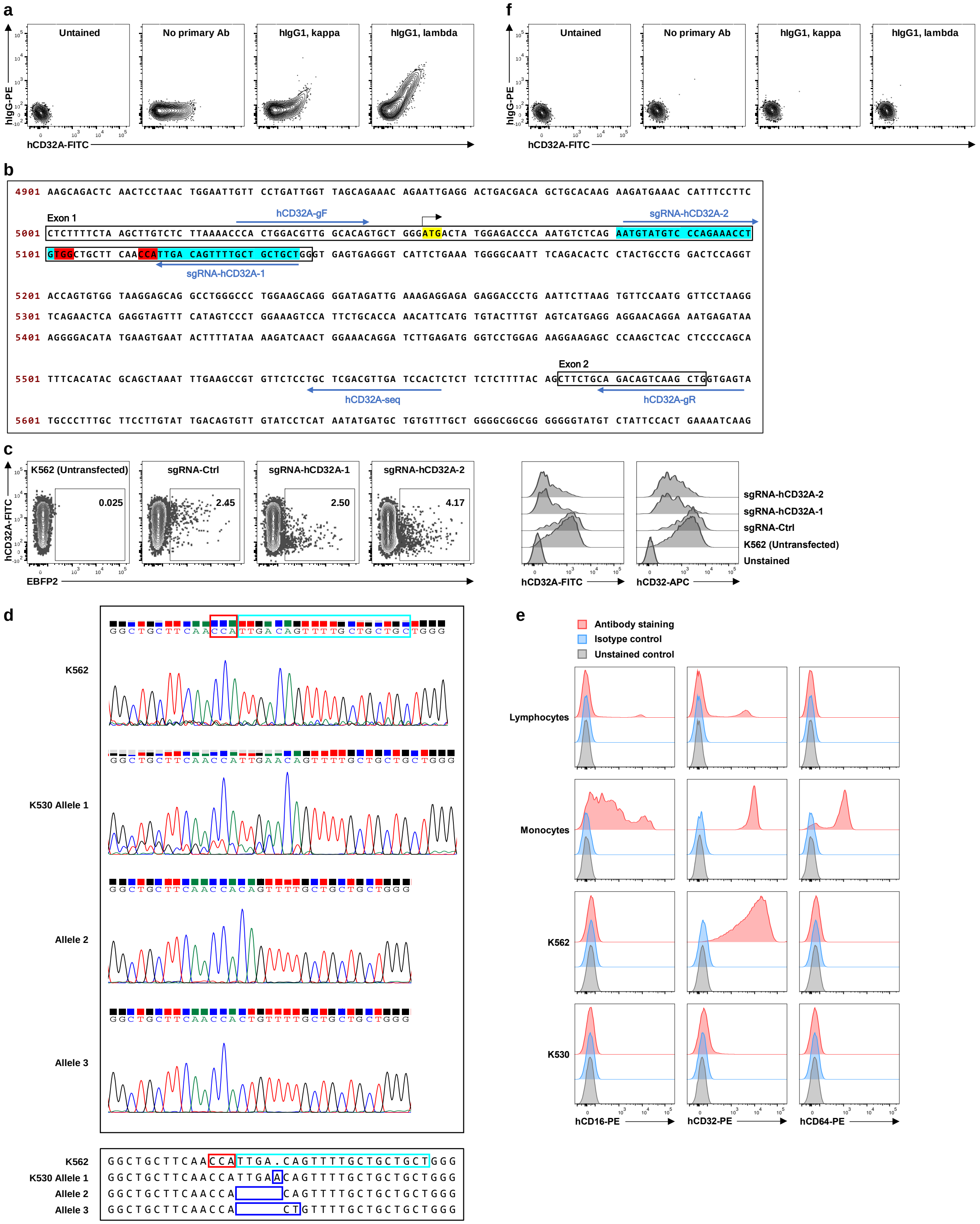

Supplementary Figure 1. The generation of K530 cell line. K562 cell line was engineered to knockout *CD32A* gene by CRISPR-Cas9 mediated gene targeting. **a**, K562 cells express Fcγ receptor CD32A and bind to human IgG1 isotype control antibodies non-specifically. **b**, The design of single guide RNAs (sgRNAs) targeting coding region in exon 1 of human *CD32A* gene

(GenBank Accession NG_012066). Exons 1 and 2 are indicated with boxes, the start codon is highlighted in yellow, sgRNA sequences are in cyan with corresponding PAM sequences in red. Primers hCD32A-gF/hCD32A-gR and hCD32A-seq were used in amplification and sequencing, respectively. **c**, Flow cytometry detection of CD32A expression at 96 hours after targeting plasmid transfection. EBFP2+ populations were gated and CD32A and CD32 expression levels were shown in histograms on the right. Total live cells of untransfected or unstained K562 cells were set as controls. **d**, Sequence analysis of targeted region in the selected monoclonal cell line K530. Three different modified alleles were identified, consistent with the triploid karyotype of K562 cell line. Cyan box shows sgRNA region, red the PAM sequence and blue the modified. **e**, Expression of Fcγ receptors CD16, CD32 and CD64 on K562 and K530 cell lines. Lymphocytes and monocytes from normal human peripheral blood mononuclear cells (PBMCs) were used as positive controls in straining. PBMCs were obtained from one healthy human subject under Duke Institutional Review Board committee guidelines. Written informed consent was obtained. **f**, As compared with K562 cells in **a**, K530 cells do not express CD32A and there's minimal non-specific binding to human IgG1 isotype control antibodies.

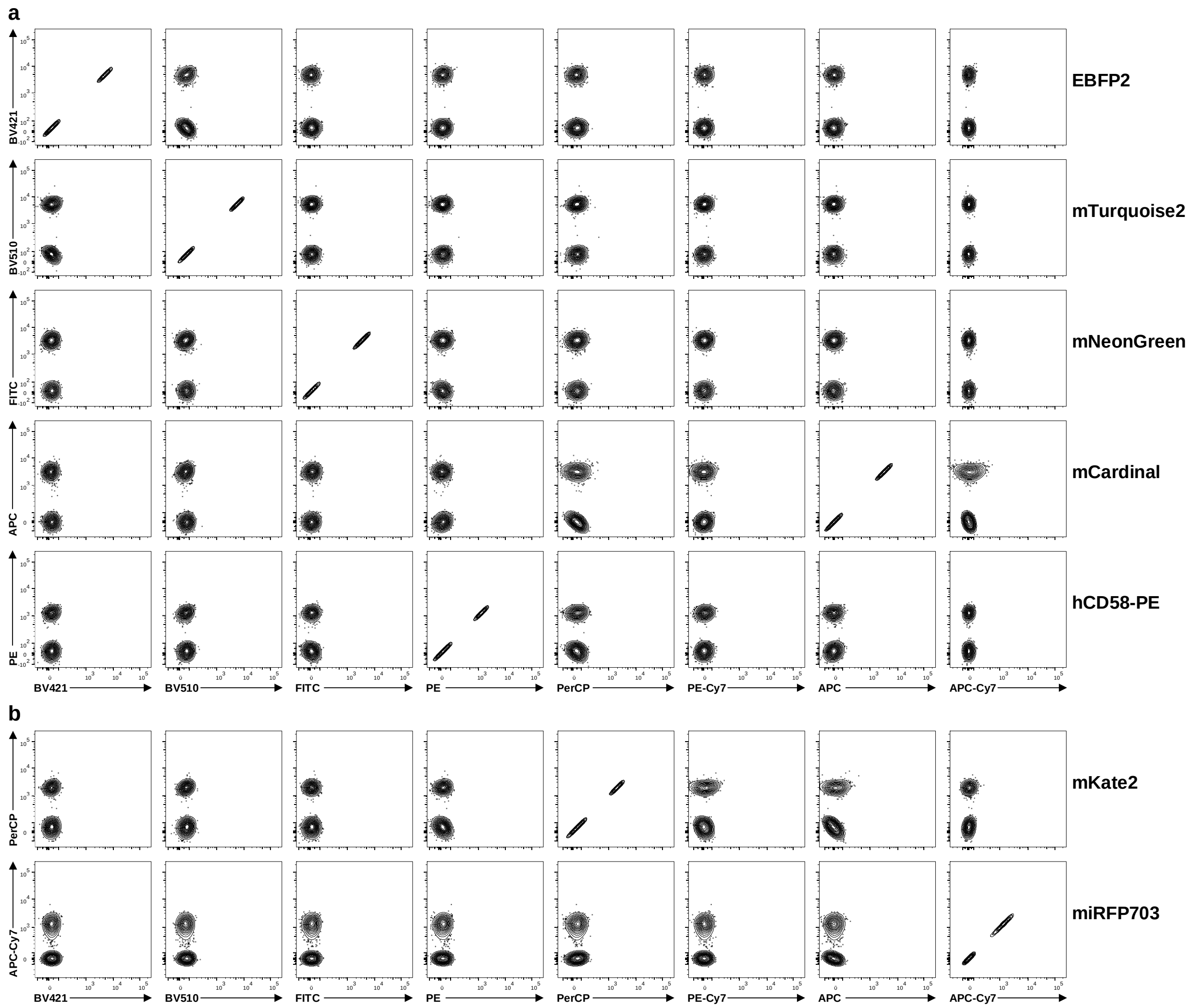

Supplementary Figure 2. FPs selected for multicolor labeling and detection on BD FACSCanto II flow cytometer. K530 cells were transduced with lentiviral vectors expressing different FPs. Monoclonal cell lines were generated and mixed with untransduced K530 cells to determine compensation settings on clinical-approved BD FACSCanto II cytometer. Those FPs with limited spreading spillovers into other channels were selected. **a**, A basic panel of FPs for four-color labeling and detection. 16 different combinations can be achieved with this basic panel. PE channel is set for the detection of analytes with PE-conjugated antibodies. For compensation settings, the endogenous human CD58 molecule was used as a surrogate analyte. **b**, An extended panel with two additional colors for detection on FACSCanto II, supporting 64 different combinations.

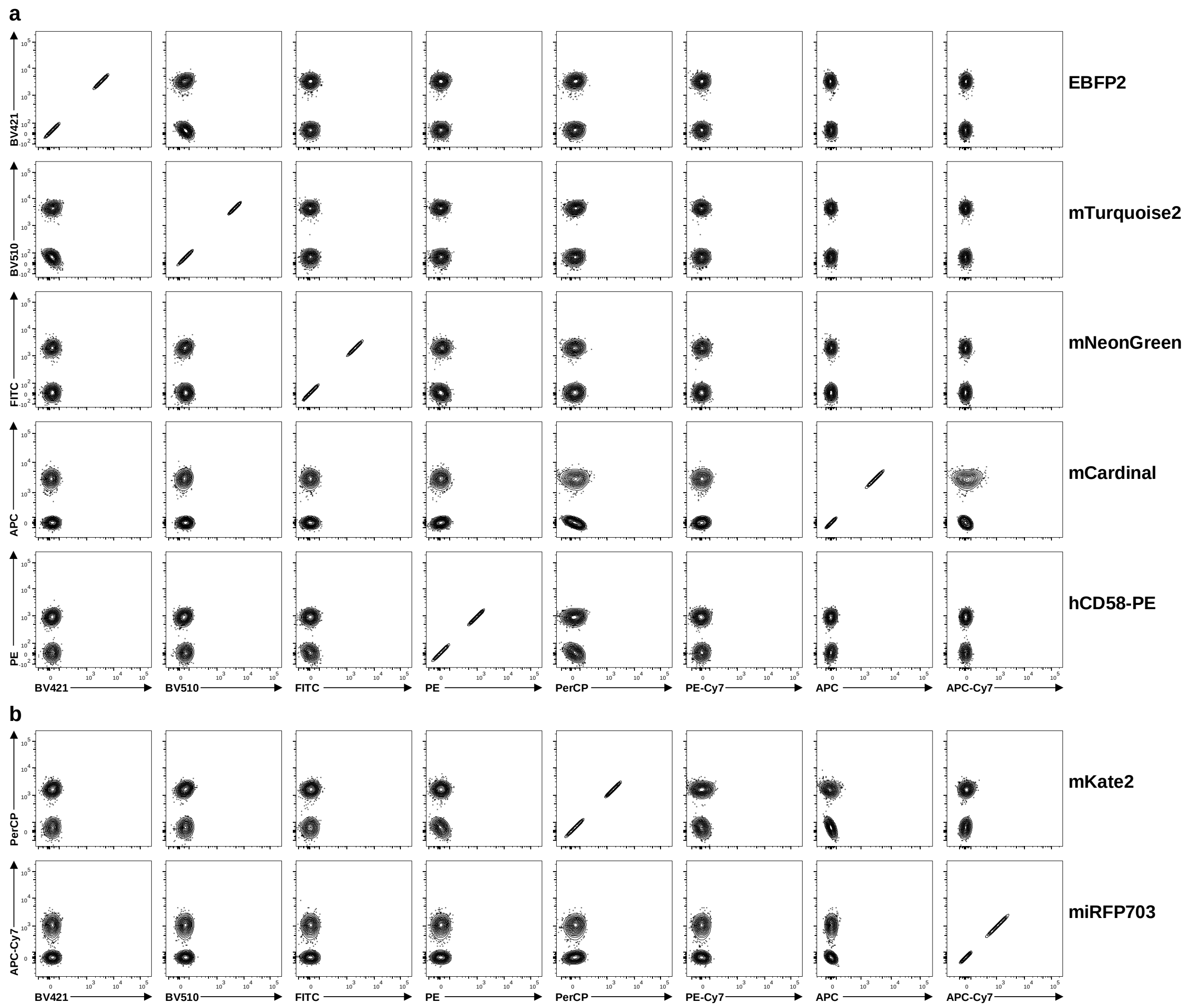

Supplementary Figure 3. Data from an independent repeat experiment for Supplementary Figure 2. FPs selected for multicolor labeling and detection on BD FACSCanto II flow cytometer. K530 cells were transduced with lentiviral vectors expressing different FPs. Monoclonal cell lines were generated and mixed with untransduced K530 cells to determine compensation settings on clinical-approved BD FACSCanto II cytometer. Those FPs with limited spreading spillovers into other channels were selected. **a**, A basic panel of FPs for four-color labeling and detection. 16 different combinations can be achieved with this basic panel. PE channel is set for the detection of analytes with PE-conjugated antibodies. For compensation settings, the endogenous human CD58 molecule was used as a surrogate analyte. **b**, An extended panel with two additional colors for detection on FACSCanto II, supporting 64 different combinations.

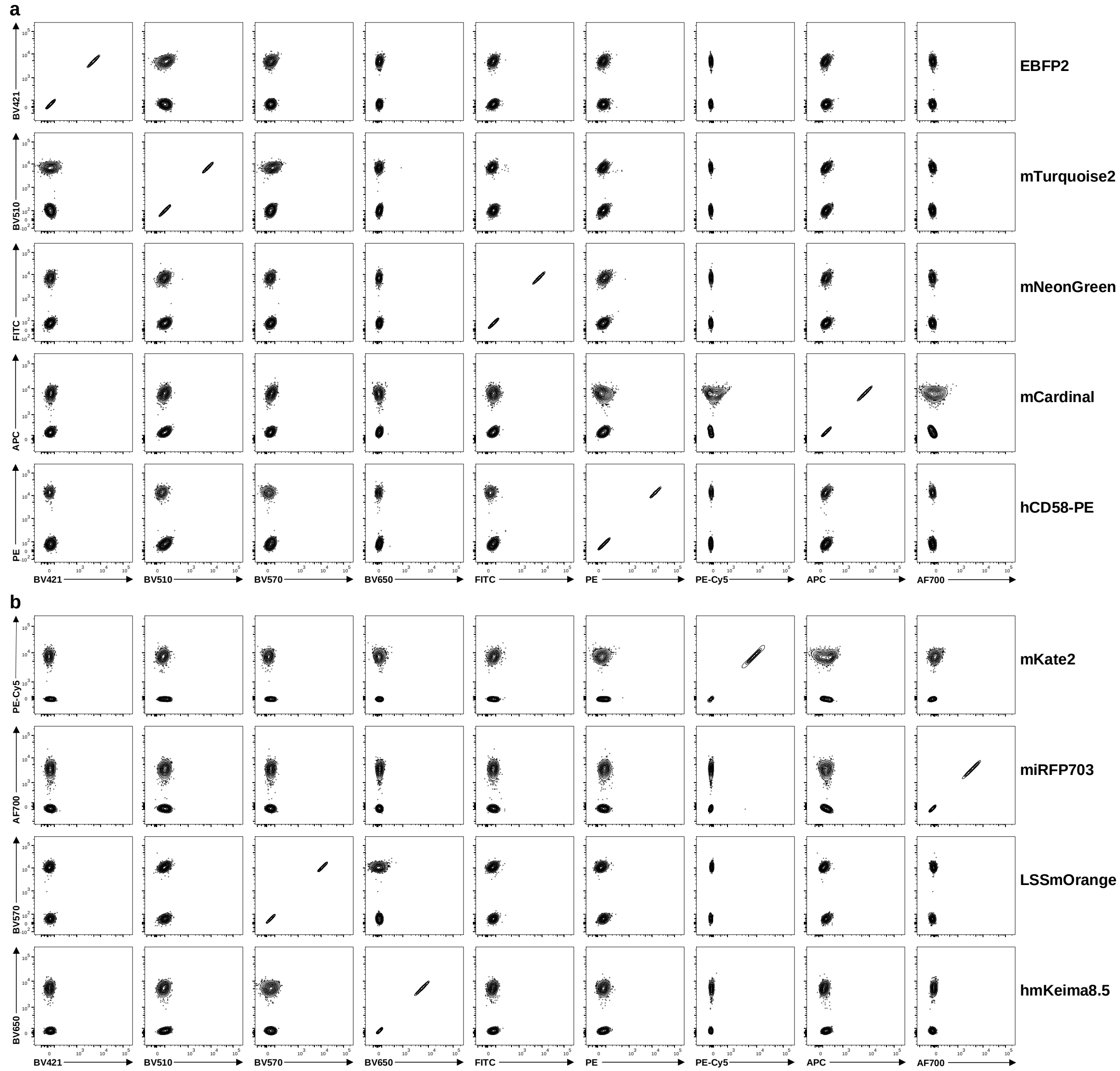

Supplementary Figure 4. FPs selected for multicolor labeling and detection on BD LSR II flow cytometer. K530 cells were transduced with lentiviral vectors expressing different FPs. Monoclonal cell lines were generated and mixed with untransduced K530 cells to determine compensation settings on BD LSR II cytometer. **a**, The same basic panel of FPs as used on Canto II cytometer (**Supplementary Figure 2**) for four-color labeling and detection. 16 different combinations can be achieved with this basic panel. PE channel is set for the detection of analytes with PE-conjugated antibodies. For compensation settings, the endogenous human CD58 molecule was used as a surrogate analyte. **b**, An extended panel with four additional colors for detection on LSR II, supporting 256 different combinations.

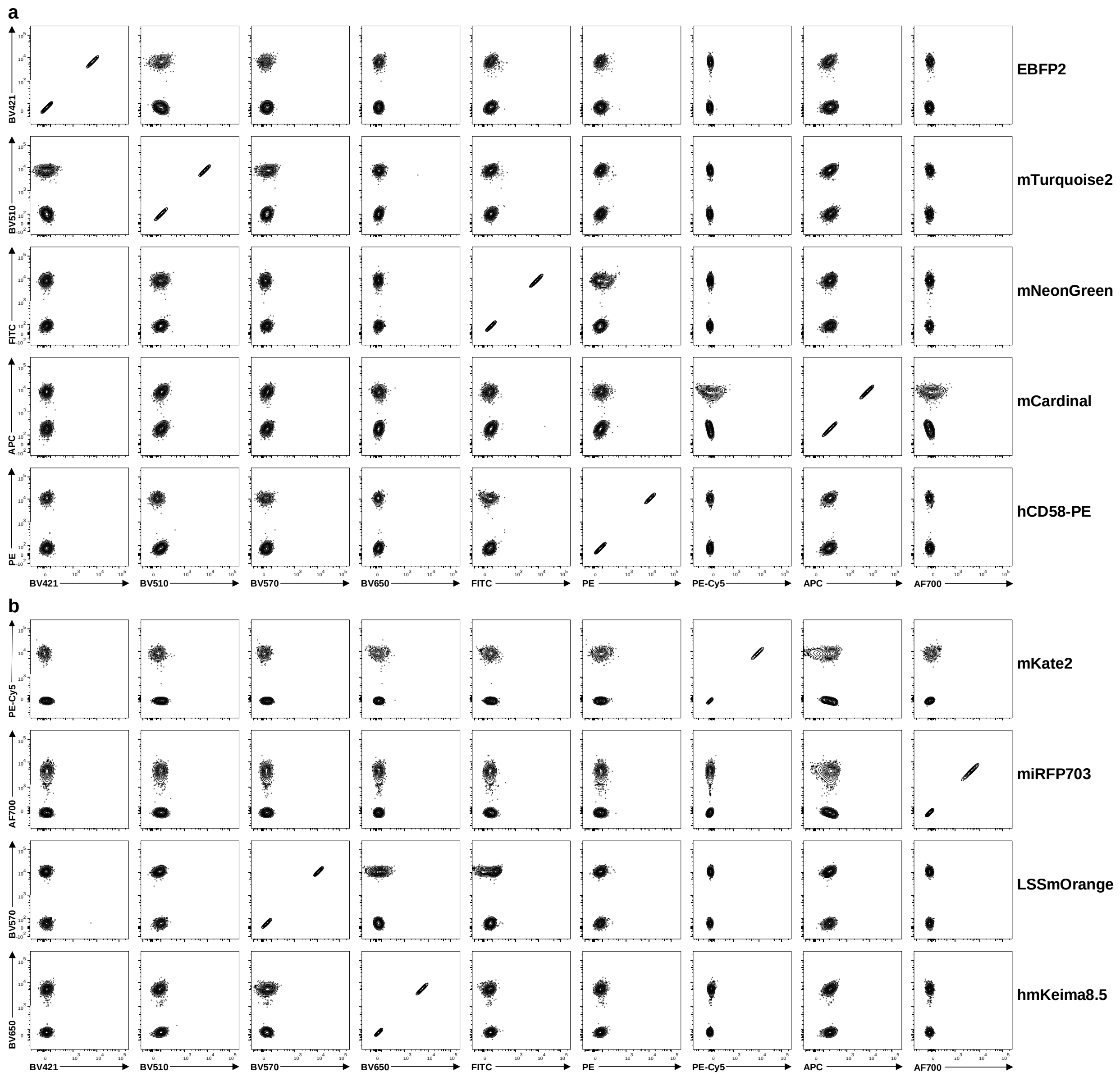

Supplementary Figure 5. Data from an independent repeat experiment for Supplementary Figure 4. FPs selected for multicolor labeling and detection on BD LSR II flow cytometer. K530 cells were transduced with lentiviral vectors expressing different FPs. Monoclonal cell lines were generated and mixed with untransduced K530 cells to determine compensation settings on BD LSR II cytometer. **a**, The same basic panel of FPs as used on Canto II cytometer (**Supplementary Figure 2**) for four-color labeling and detection. 16 different combinations can be achieved with this basic panel. PE channel is set for the detection of analytes with PE-conjugated antibodies. For compensation settings, the endogenous human CD58 molecule was used as a surrogate analyte. **b**, An extended panel with four additional colors for detection on LSR II, supporting 256 different combinations.

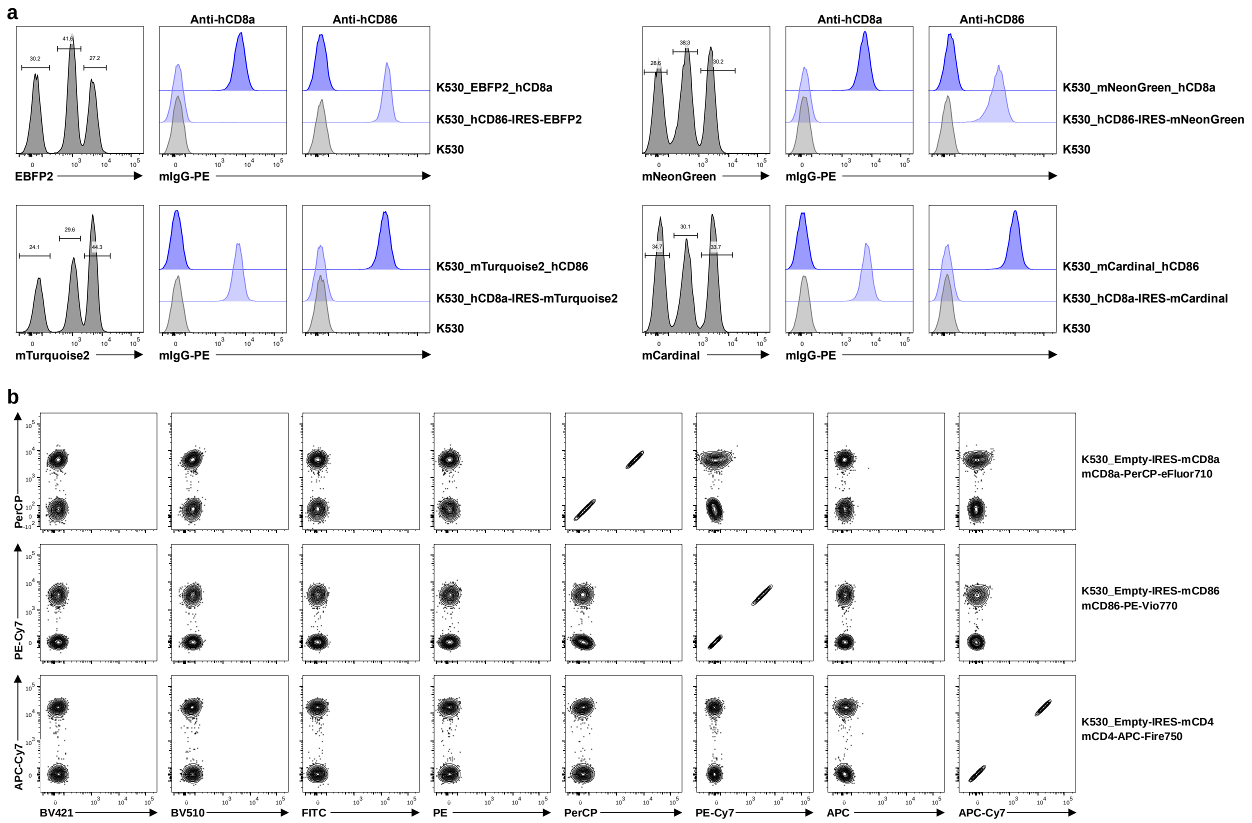

Supplementary Figure 6. Alternative strategies to increase multiplicity of labeling and detection on BD FACSCanto II flow cytometer. a, Differential labeling with the same FPs. Three types of monoclonal cell lines were generated for each of the four FPs in the basic panel in **Supplementary Figure 2**: 1) untransduced K530 cells, 2) K530 cells transduced to express an example analyte (human CD8a or CD86) along with an FP following the IRES (internal ribosome entry site) bicistronic element (hCD8a/hCD86-IRES-FP), and 3) K530 cells transduced to express a different example analyte (human CD86 or CD8a) and the same FP as above via separate lentiviral vectors. These three types of cell lines were pooled and detected with FACSCanto II. Three histograms were detected in corresponding fluorescence channel for each FP. The surface expression of human CD8a and CD86 were plotted for each population in FP channel, with FP-negative population shown in gray, FP-intermediate population in light blue and FP-high population in dark blue. The expression pattern of human CD8a and CD86 was consistent with the designation of these three populations as shown on the right of histogram plots, indicating that IRES-driven expression of FPs resulted in 5- to 7-fold less fluorescent intensity as compared with that by direct driven with the same promoter, allowing a good separation of pooled K530 cell lines expressing the same FP by protomer-driven and IRES-driven. With this alternative strategy, the multiplexity can be increased from 16-plex to 48-plex without the need to expand the basic reporter cell line panel. **b**, Co-expression of a non-relevant surface marker along with analyte protein with IRES bicistronic vectors. The co-expressed surface marker can then be detected in additional fluorescence channels. The multiplexity can be increased from 16-plex to 64-plex without the need to expand the basic reporter cell line panel.

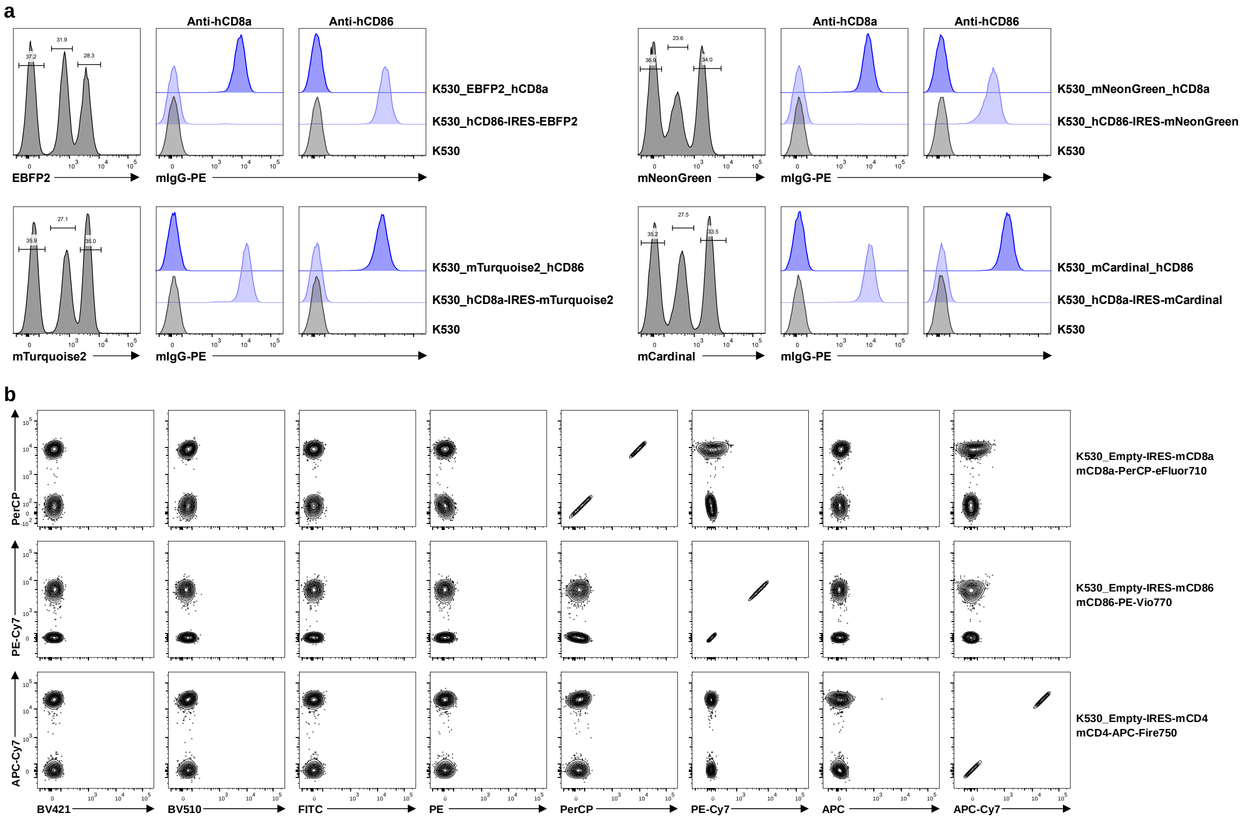

Supplementary Figure 7. Data from an independent repeat experiment for Supplementary Figure 6. Alternative strategies to increase multiplicity of labeling and detection on BD FACSCanto II flow cytometer. **a**, Differential labeling with the same FPs. Three types of monoclonal cell lines were generated for each of the four FPs in the basic panel in **Supplementary Figure 2**: 1) untransduced K530 cells, 2) K530 cells transduced to express an example analyte (human CD8a or CD86) along with an FP following the IRES (internal ribosome entry site) bicistronic element (hCD8a/hCD86-IRES-FP), and 3) K530 cells transduced to express a different example analyte (human CD86 or CD8a) and the same FP as above via separate lentiviral vectors. These three types of cell lines were pooled and detected with FACSCanto II. Three histograms were detected in corresponding fluorescence channel for each FP. The surface expression of human CD8a and CD86 were plotted for each population in FP channel, with FP-negative population shown in gray, FP-intermediate population in light blue and FP-high population in dark blue. The expression pattern of human CD8a and CD86 was consistent with the designation of these three populations as shown on the right of histogram plots, indicating that IRES-driven expression of FPs resulted in 5- to 7-fold less fluorescent intensity as compared with that by direct driven with the same promoter, allowing a good separation of pooled K530 cell lines expressing the same FP by protomer-driven and IRES-driven. With this alternative strategy, the multiplexity can be increased from 16-plex to 48-plex without the need to expand the basic reporter cell line panel. **b**, Co-expression of a non-relevant surface marker along with analyte protein with IRES bicistronic vectors. The co-expressed surface marker can then be detected in additional fluorescence channels. The multiplexity can be increased from 16-plex to 64-plex without the need to expand the basic reporter cell line panel.

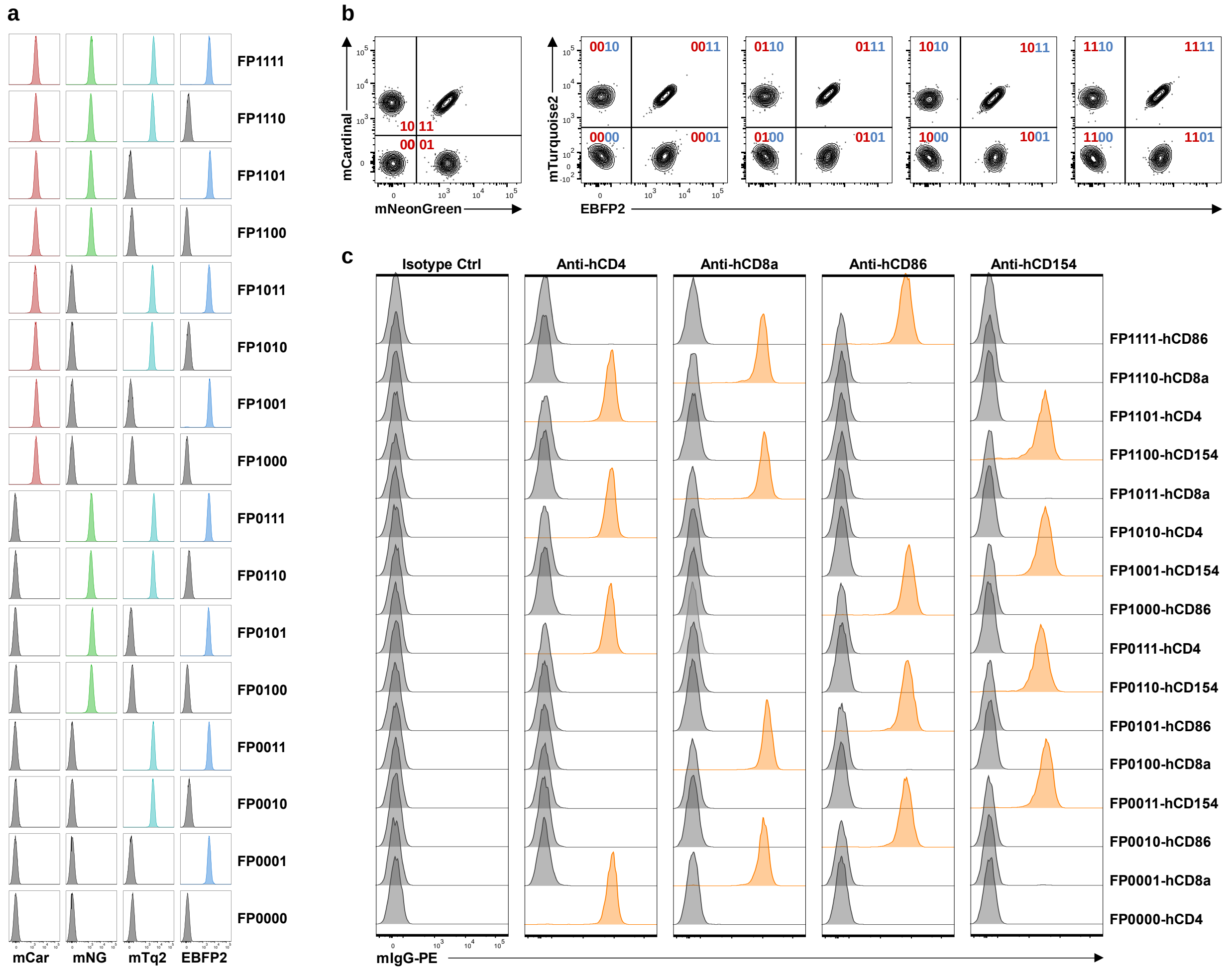

Supplementary Figure 8. Data from an independent repeat experiment for Figure 1. A multiplex immunoassay based on FP-barcoded reporter cell lines. **a**, A basic panel of FP-barcoded reporter cell lines. K530 cells were transduced with different combinations of four FPs to produce 16 uniquely FP-barcoded reporter cell lines. The absence/presence of fluorescence from FPs EBFP2, mTurquoise2 (mTq2), mNeonGreen (mNG) and mCardinal (mCar) are designated as four digits of binary barcodes as shown on the right of histograms for each individual cell line. **b**, Demultiplexing of pooled FP-barcoded reporter cell lines by flow cytometry. **c**, The 16 barcoded reporter cell lines were transduced to express human CD4, CD8a, CD86 and CD154 molecules in a shifted pattern relative to FP expression. These cells were pooled and stained with corresponding mouse monoclonal antibodies (as indicated on the top of each histogram) followed by a PE-conjugated anti-mouse IgG antibody. Signals from individual reporter cell lines were demultiplexed as shown in **b**, and the binding by corresponding antibodies were plotted as half-offset histograms. In all cases, the detected expression patterns were consistent with antigen expression by barcoded cells before multiplexing as shown on the right of histograms for each individual cell line. Isotype Ctrl, mouse IgG1, kappa isotype control antibody.

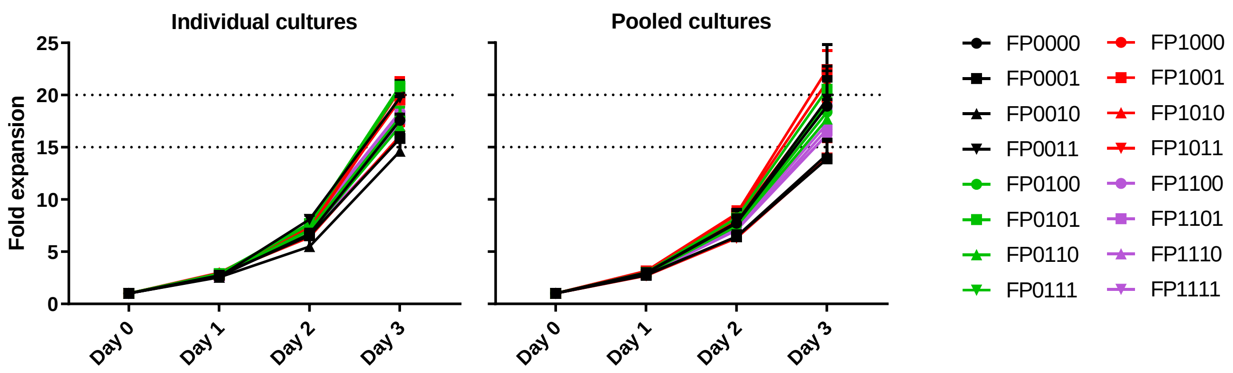

Supplementary Figure 9. Growth rates of the 16 FP-barcoded reporter cell lines. The reporter cell lines were cultured individually (left panel) or pooled at equal ratios (right panel) and sampled daily for cell counting with flow cytometry using CountBright beads as reference. Duplicate wells of individual cultures and triplicate pooled cultures were counted at each time point. Bars indicate mean + SD.

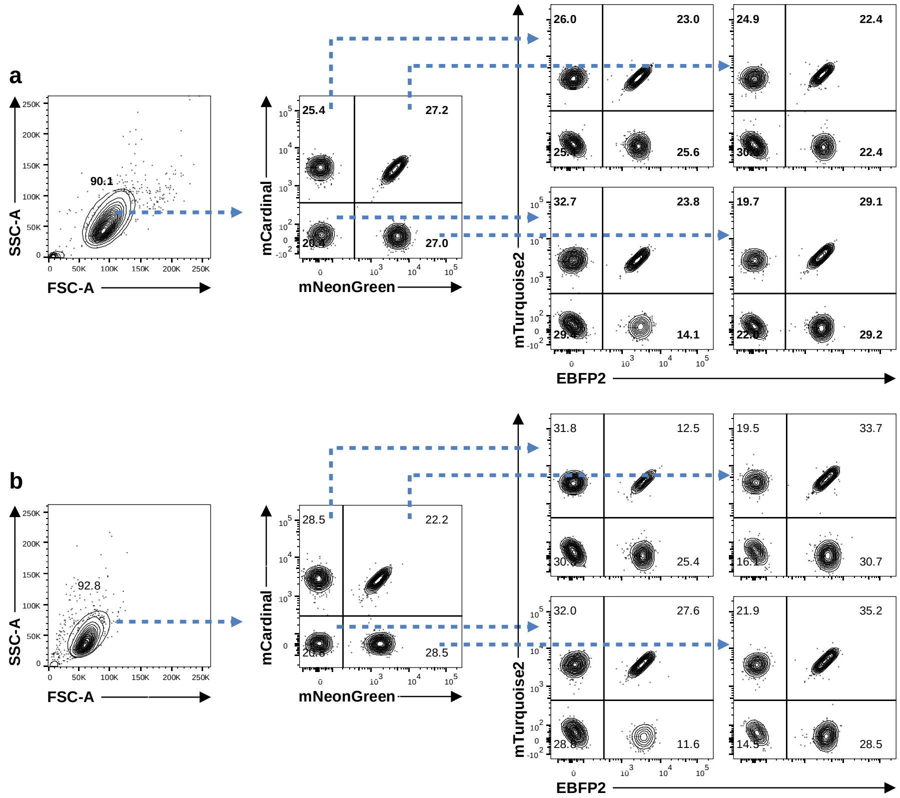

Supplementary Figure 10. Gating strategy for demultiplexing of pooled FP-barcoded reporter cell lines. a and **b**, Data from two independent repeat experiments. Total live cells were gated and plotted for FPs mNeonGreen (mNG) and mCardinal (mCar) expression, resulting in four distinct populations: $mNG^{-}mCar^{-}$, $mNG^{-}mCar^{+}$, $mNG^{+}mCar^{-}$, $mNG^{+}mCar^{+}$. For each population, the expression of EBFP2 and mTurquoise2 (mTq2) were further plotted, yielding four well-separated sub-populations: $EBFP2^{-}mTq2^{-}$, $EBFP2^{+}mTq2^{-}$, $EBFP2^{-}mTq2^{+}$ and $EBFP2^{+}mTq2^{+}$. These 16 subpopulations represent the 16 individual reporter cell lines before pooling. Antibody binding to each subpopulation can then be analyzed by gating on each of them. **a**, The same data set was used from **Figure 1c**, isotype control stained sample. **b**, The same data set was used from **Supplementary Figure S8c**, isotype control stained sample.

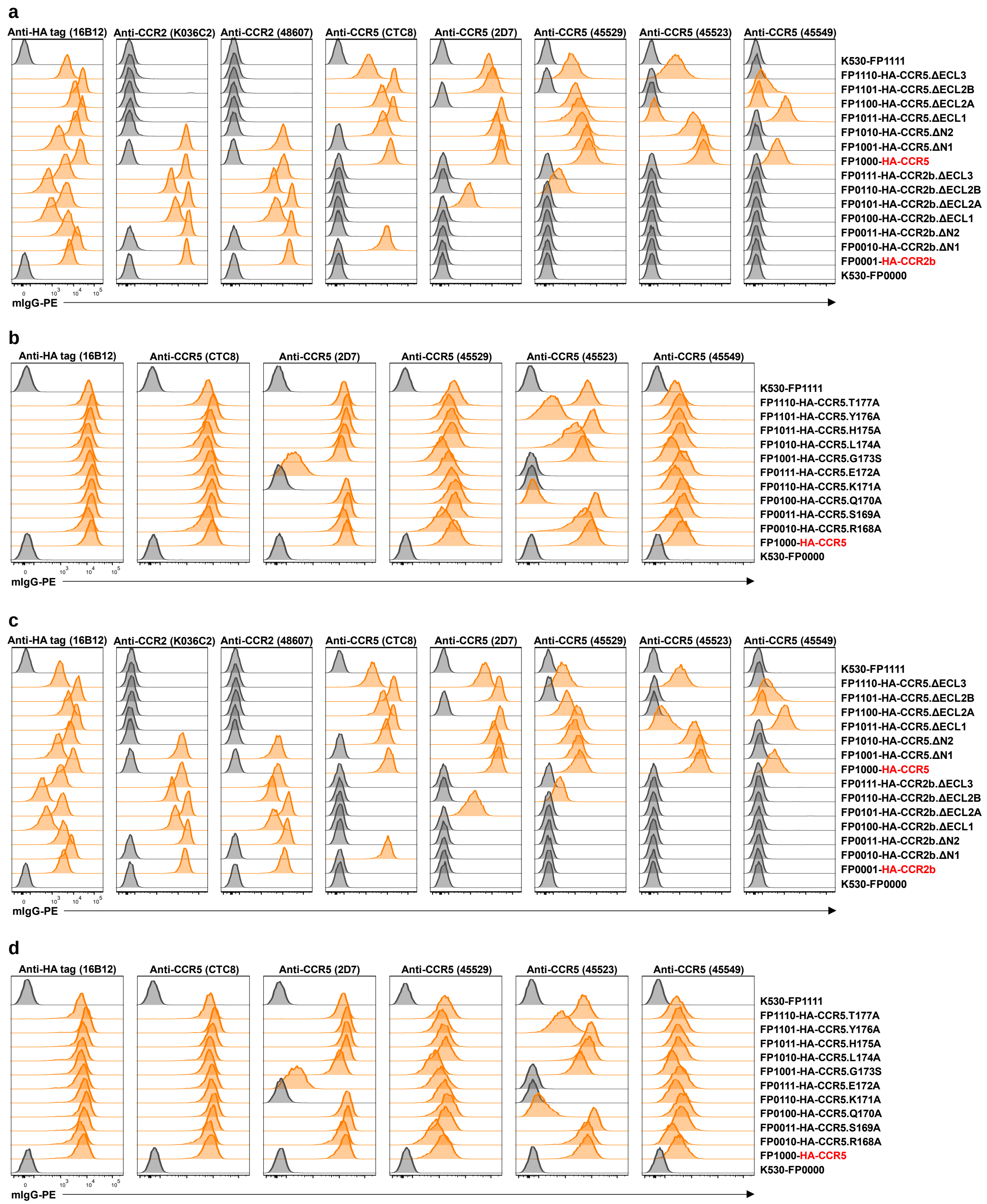

Supplementary Figure 11. Data from two independent repeat experiments for Figure 3. Application of the multiplex immunoassay in domain/epitope mapping of antibodies specific for human CCR2 and CCR5. **a** and **b**, Data from one independent repeat experiment. **c** and **d**, Data from another independent repeat experiment. **a** and **c**, corresponding to **Figure 3b**. Domain mapping with multiplexed reporter cell lines expressing CCR2b and CCR5 domain-swapped mutants. The binding to each reporter cell line was demultiplexed as shown in **Figure 1**. Histograms with MFI values above 2-fold of background (the average MFI value of internal

control cell lines K530-FP0000 and K530-FP1111) were scored as positive and highlighted in orange. MFI values for individual histograms are listed in **Supplementary Tables 3**. **b** and **d**, corresponding to **Figure 3d**. Epitope mapping with multiplexed reporter cell lines expressing CCR5 ECL2A point mutants. The data were analyzed in the same way as in **a** and **c**. MFI values for individual histograms are listed in **Supplementary Tables 4**.

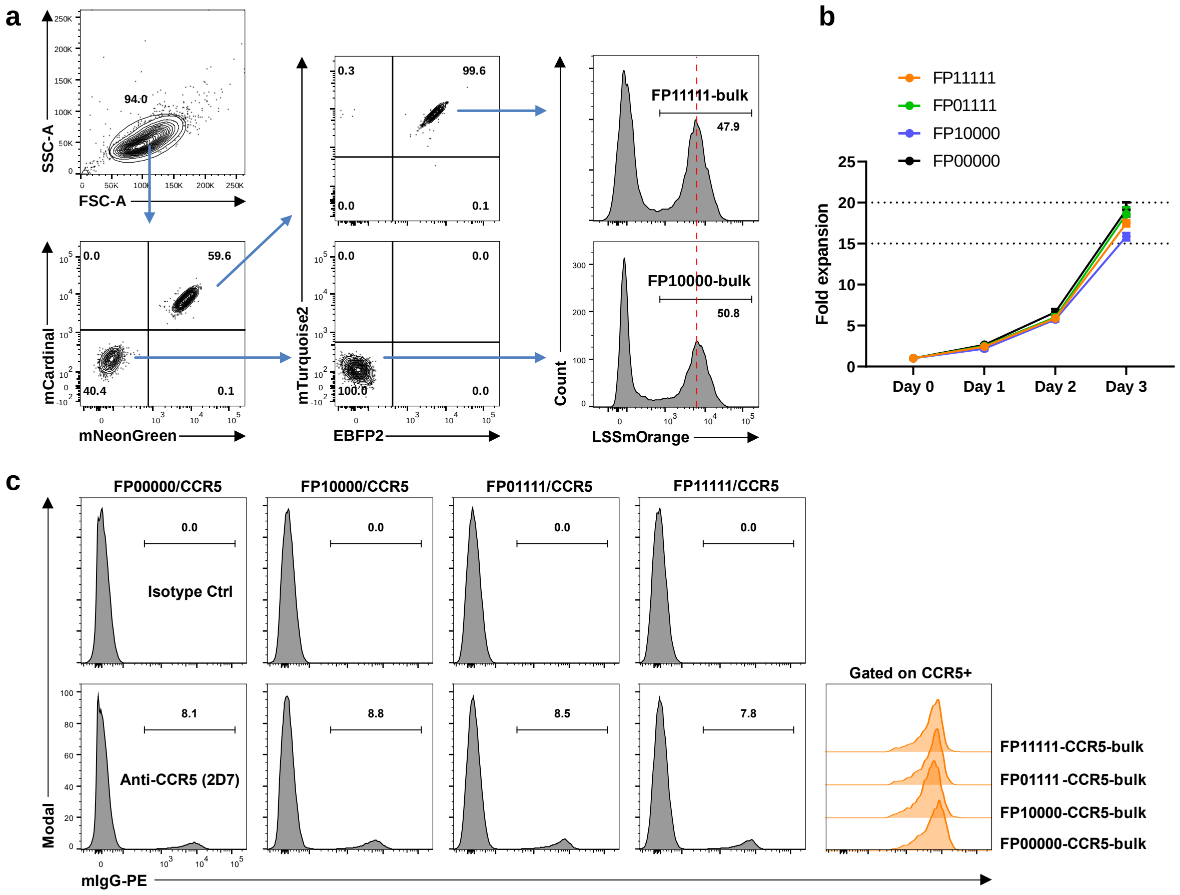

Supplementary Figure 12. Expanding the 16-plex reporter cell line panel by introducing a fifth FP. Reporter cell lines FP0000 and FP1111, with none or four preexisting FPs, were pooled and transduced to express LSSmOrange. **a**, Gating strategy of the bulk culture after transduction. Similar transduction efficiencies and LSSmOrange intensities were achieved in both parental lines. **b**, Growth rates of monoclonal cell lines. Duplicate wells of individually cultured cells were sampled daily for cell counting with flow cytometry using CountBright beads as reference. Bars indicates mean + SD. **c**, The four reporter cell lines generated above were transduced to express human CCR5. Surface expression of CCR5 was detected 3 days after transduction without antibiotic selection. Bulk transductants were stained with either an isotype control antibody (mouse IgG2a, kappa) or anti-CCR5 (clone 2D7) followed by a PE-conjugated anti-mouse IgG secondary antibody. CCR5+ populations were gated and plotted in an overlaid histogram on the right.

Supplementary Table 1 FPs selected for multi-color barcoding of reporter cell lines

Fluorescent protein	λ_{ex} (nm)	λ_{em} (nm)	ε (M^{-1} cm^{-1})	φ	Brightness	Ref.	BD FACSCanto II Settings			BD LSR II Settings		
							Laser (nm)	Channel	Mirror, Filter	Laser (nm)	Channel	Mirror, Filter
EBFP2	383	448	32,000	0.56	17.9	1	405	BV421	NA, 450/50	405	BV421	NA, 450/50
mTurquoise2	434	474	30,000	0.93	27.9	2	405	BV510	502LP, 510/50	405	BV510	505LP, 515/20
mNeonGreen	506	517	116,000	0.80	92.8	3	488	FITC	502LP, 530/30	488	FITC	495LP, 525/50
mCardinal	604	659	87,000	0.19	17.0	4	633	APC	NA, 660/20	639	APC	NA, 670/30
mKate2	588	633	62,500	0.40	24.9	5	488	PerCP	655LP, 670LP	532	PE-Cy5	655LP, 660/20
miRFP703	674	703	90,900	0.086	7.8	6	633	APC-Cy7	735LP, 780/60	639	AF700	690LP, 730/45
LSSmOrange	437	572	52,000	0.45	23.4	7	—	—	—	405	BV570	555LP, 560/40
hmKeima8.5	438	612	32,000	0.34	10.9	8	—	—	—	405	BV650	635LP, 670/30

λ_{ex}, excitation maximum. λ_{em}, emission maximum. ε, extinction coefficient. φ, fluorescence quantum yield.

References

1. Ai, H.W., Shaner, N.C., Cheng, Z., Tsien, R.Y. & Campbell, R.E. Exploration of new chromophore structures leads to the identification of improved blue fluorescent proteins. *Biochemistry* **46**, 5904-5910 (2007).
2. Goedhart, J. et al. Structure-guided evolution of cyan fluorescent proteins towards a quantum yield of 93%. *Nat Commun* **3**, 751 (2012).
3. Shaner, N.C. et al. A bright monomeric green fluorescent protein derived from Branchiostoma lanceolatum. *Nat Methods* **10**, 407-409 (2013).
4. Chu, J. et al. Non-invasive intravital imaging of cellular differentiation with a bright red-excitable fluorescent protein. *Nat Methods* **11**, 572-578 (2014).
5. Shcherbo, D. et al. Far-red fluorescent tags for protein imaging in living tissues. *Biochem J* **418**, 567-574 (2009).
6. Shcherbakova, D.M. et al. Bright monomeric near-infrared fluorescent proteins as tags and biosensors for multiscale imaging. *Nat Commun* **7**, 12405 (2016).
7. Shcherbakova, D.M., Hink, M.A., Joosen, L., Gadella, T.W. & Verkhusha, V.V. An orange fluorescent protein with a large Stokes shift for single-excitation multicolor FCCS and FRET imaging. *J Am Chem Soc* **134**, 7913-7923 (2012).
8. Guan, Y. et al. Live-cell multiphoton fluorescence correlation spectroscopy with an improved large Stokes shift fluorescent protein. *Mol Biol Cell* **26**, 2054-2066 (2015).

Supplementary Table 2 MFI values of individual histograms shown in Figure 2

	Barcode	FP0000	FP0001	FP0010	FP0011	FP0100	FP0101	FP0110	FP1000	FP1001	FP1010	FP1100	FP1101	FP1110	FP1111
	Antigen	—	H1.SI06	H2.JP57	H4.NB10	H1.CH10	H8.CA07	H5.VN04	H3.HK68	H14.WI10	H10.JX13	H6.TW13	H7.TW17	H3.TX12	—
Expt.1 (dark grey or dark orange shaded)	FI6	58	21238	4090	10676	2339	12035	2509	3130	1760	1200	4522	699	2227	59
	S5V2-29	49	230	1454	8951	5110	617	75	7893	1052	161	67	132	2069	51
	CH67	36	14687	40	43	40	36	40	39	42	41	38	38	36	39
	HC19	41	47	44	53	41	45	47	20872	57	54	41	41	41	43
	hIgG1K	34	32	37	39	34	33	39	36	37	40	38	36	36	39
	hIgG1L	33	31	36	39	32	32	38	35	34	39	36	35	35	38
Expt.2 (light grey or light orange shaded)	FI6	71	21818	3873	13004	2024	11402	2796	3141	2176	1322	4028	692	2314	70
	S5V2-29	65	249	1975	10411	5065	669	112	8539	1372	231	85	168	2609	65
	CH67	42	15859	46	45	54	45	44	43	51	43	44	44	39	40
	HC19	50	58	95	72	56	69	66	21697	97	98	54	50	59	49
	hIgG1K	42	37	43	43	40	40	43	43	41	41	43	42	39	39
	hIgG1L	41	36	41	41	36	37	40	41	39	40	42	40	38	37

For each sample, MFI values above 2-fold of the average MFI of internal control cell lines (FP0000 and FP1111) were scored as positive and highlighted in orange. Green highlighted indicates a correction, with major peak of the histogram (accounts for 95% of total events) gated for MFI calculation, to avoid non-specific binding events due to a potential technical issue.

Supplementary Table 3 MFI values of individual histograms shown in Figures 3b, S11a and S11c

	Barcode	FP0000	FP0001	FP0010	FP0011	FP0100	FP0101	FP0110	FP0111	FP1000	FP1001	FP1010	FP1011	FP1100	FP1101	FP1110	FP1111
	Antigen	—	CCR2b domain mutants							CCR5 domain mutants							—
			WT	ΔN1	ΔN2	ΔECL1	ΔECL2A	ΔECL2B	ΔECL3	WT	ΔN1	ΔN2	ΔECL1	ΔECL2A	ΔECL2B	ΔECL3	
Fig.3b	16B12	38	3443	8073	3539	611	3087	370	2786	9158	2028	7513	12907	5870	13475	2328	24
	K036C2	40	19847	32	25764	7353	27337	4910	14963	14	15950	13	24	24	33	20	24
	48607	44	16803	38	21780	5462	26341	4661	8888	21	9067	18	24	30	37	25	27
	CTC8	44	44	9999	39	48	47	51	44	10651	20	7972	17579	6024	16712	1663	27
	2D7	40	40	33	36	43	962	48	42	20715	26947	16324	26496	30	10968	5615	26
	45529	37	39	30	33	44	41	520	38	4049	3704	2757	2559	1095	31	745	22
	45523	40	42	33	34	44	43	45	40	13897	16109	8846	131	28	35	1460	24
	45549	40	43	36	40	52	45	44	44	621	23	21	883	97	106	29	23
Fig.S11a	16B12	37	6109	13122	4861	884	4253	597	3675	19902	1853	11913	23142	11253	24793	4574	33
	K036C2	38	25171	48	29917	7301	30200	4321	21836	30	22737	41	58	37	54	49	35
	48607	36	20050	42	23350	4529	26502	3468	9651	30	8514	37	39	32	41	42	31
	CTC8	35	31	8143	37	38	32	41	37	12718	35	5771	17678	5349	18905	1234	29
	2D7	31	30	37	39	37	750	40	37	25377	29779	15363	26596	27	8070	6466	25
	45529	34	32	34	32	29	24	182	28	4279	3491	2097	1426	1028	25	712	24
	45523	32	30	31	30	29	24	31	29	11099	12128	3920	69	24	27	596	22
	45549	28	28	27	33	30	24	32	29	492	29	28	1126	53	119	28	22
Fig.S11c	16B12	45	3109	6974	2997	513	2712	344	2071	8394	1642	6448	12309	5213	14068	2064	49
	K036C2	47	20216	43	25821	6980	26968	4566	13058	47	13432	43	44	57	56	54	58
	48607	46	10711	44	14223	3635	16451	2737	4995	51	5047	43	44	53	55	50	50
	CTC8	48	46	9198	49	47	46	49	46	10290	54	7746	17451	5596	18263	1737	47
	2D7	45	46	42	46	41	1258	43	38	16813	21556	11676	23993	45	18964	4031	41
	45529	44	44	42	45	42	41	201	42	1434	1313	875	1205	417	47	297	41
	45523	44	44	42	46	47	46	49	46	7592	7800	4342	195	52	59	953	46
	45549	53	52	50	61	60	56	58	54	353	67	58	953	101	203	57	48

For each sample, MFI values above 2-fold of the average MFI of internal control cell lines (FP0000 and FP1111) were scored as positive and highlighted in orange.

Supplementary Table 4 MFI values of individual histograms shown in Figures 3d, S11b and S11d

Barcode		FP0000	FP1000	FP0010	FP0011	FP0100	FP0110	FP0111	FP1001	FP1010	FP1011	FP1101	FP1110	FP1111
Antigen		—	CCR5 ECL2A point mutants											—
			WT	R168A	S169A	Q170A	K171A	E172A	G173S	L174A	H175A	Y176A	T177A	
Fig.3d	16B12	33	7148	5281	8126	9268	8291	6564	5834	6363	7138	10292	5681	17
	CTC8	42	8911	6126	10626	11932	9500	9085	7379	7717	9673	13579	7455	24
	2D7	37	18531	12810	22181	20556	48	216	10679	19114	19573	20908	13887	21
	45529	38	3150	1202	3287	3829	2669	1881	1180	3083	2660	2455	2794	23
	45523	36	10857	6427	17390	133	43	42	5684	5639	13535	760	7688	21
	45549	38	561	285	637	697	379	576	268	475	465	515	383	22
Fig.S11b	16B12	42	13645	9042	14195	14022	14794	11605	9815	9679	12299	15291	10266	32
	CTC8	38	8170	4531	8544	8194	7898	6999	5344	5038	7125	9681	5601	28
	2D7	37	18264	12321	20392	16769	42	209	10951	14861	17594	18679	13916	28
	45529	38	3311	1227	3816	3848	2995	2259	1141	2852	2813	2310	3365	29
	45523	31	7541	3334	13044	57	36	33	4080	1795	9787	296	6352	25
	45549	33	466	234	510	483	284	473	198	384	370	388	270	25
Fig.S11d	16B12	43	5870	4415	6556	7724	6722	5411	4872	4919	6147	8376	4812	38
	CTC8	48	7075	5384	8835	9472	7943	7617	6265	6339	8194	10947	6355	39
	2D7	48	14748	11372	19995	17955	52	244	9332	16386	17847	18255	12324	38
	45529	44	1288	657	1706	1847	1434	1015	616	1559	1429	1152	1520	32
	45523	47	5564	4876	11455	133	44	42	3207	5894	8431	712	4461	35
	45549	51	349	217	450	461	278	384	218	358	354	358	296	36

For each sample, MFI values above 2-fold of the average MFI of internal control cell lines (FP0000 and FP1111) were scored as positive and highlighted in orange.
