# Supplementary material for: A cell-based multiplex immunoassay platform using fluorescent protein-barcoded reporter cell lines
Source: Commun Biol. 2021 Nov 25;4:1338. doi: 10.1038/s42003-021-02881-w (PMC8617053; doi:10.1038/s42003-021-02881-w)
Supplement: Supplementary file 2 — Reporting Summary [file 42003_2021_2881_MOESM2_ESM.pdf]

nature portfolio

Corresponding author(s): Garnett Kelsoe

Last updated by author(s): Oct 25, 2021

Reporting Summary

Nature Portfolio wishes to improve the reproducibility of the work that we publish. This form provides structure for consistency and transparency in reporting. For further information on Nature Portfolio policies, see our Editorial Policies and the Editorial Policy Checklist.

Statistics

For all statistical analyses, confirm that the following items are present in the figure legend, table legend, main text, or Methods section.

n/a	Confirmed	
☒	☐	The exact sample size (*n*) for each experimental group/condition, given as a discrete number and unit of measurement
☒	☐	A statement on whether measurements were taken from distinct samples or whether the same sample was measured repeatedly
☒	☐	The statistical test(s) used AND whether they are one- or two-sided<br>*Only common tests should be described solely by name; describe more complex techniques in the Methods section.*
☒	☐	A description of all covariates tested
☒	☐	A description of any assumptions or corrections, such as tests of normality and adjustment for multiple comparisons
☒	☐	A full description of the statistical parameters including central tendency (e.g. means) or other basic estimates (e.g. regression coefficient) AND variation (e.g. standard deviation) or associated estimates of uncertainty (e.g. confidence intervals)
☒	☐	For null hypothesis testing, the test statistic (e.g. *F*, *t*, *r*) with confidence intervals, effect sizes, degrees of freedom and *P* value noted<br>*Give P values as exact values whenever suitable.*
☒	☐	For Bayesian analysis, information on the choice of priors and Markov chain Monte Carlo settings
☒	☐	For hierarchical and complex designs, identification of the appropriate level for tests and full reporting of outcomes
☒	☐	Estimates of effect sizes (e.g. Cohen's *d*, Pearson's *r*), indicating how they were calculated

Our web collection on statistics for biologists contains articles on many of the points above.

Software and code

Policy information about availability of computer code

Data collection	BD FACSDiva (Version 9.0)
Data analysis	FlowJO (Version 10.7.2), Chromas (Version 2.6.6) and Microsoft Office (Version 1808)

For manuscripts utilizing custom algorithms or software that are central to the research but not yet described in published literature, software must be made available to editors and reviewers. We strongly encourage code deposition in a community repository (e.g. GitHub). See the Nature Portfolio guidelines for submitting code & software for further information.

Data

Policy information about availability of data

All manuscripts must include a data availability statement. This statement should provide the following information, where applicable:

- Accession codes, unique identifiers, or web links for publicly available datasets
- A description of any restrictions on data availability
- For clinical datasets or third party data, please ensure that the statement adheres to our policy

All raw data generated during this project are available from the authors upon request. Licensing from Allele Biotechnology and Pharmaceuticals Inc. is needed prior to material transfer of plasmids containing mNeonGreen sequences. All other plasmids are available from the authors upon request.

Field-specific reporting

Please select the one below that is the best fit for your research. If you are not sure, read the appropriate sections before making your selection.

☒ Life sciences ☐ Behavioural & social sciences ☐ Ecological, evolutionary & environmental sciences

For a reference copy of the document with all sections, see nature.com/documents/nr-reporting-summary-flat.pdf

Life sciences study design

All studies must disclose on these points even when the disclosure is negative.

Sample size	For flow cytometry analysis of K562 derivative cell lines, >10,000 cells for a single cell line or >3,000 cells for each individual cell line in pooled cell samples were acquired. With clonal cell lines, these sample sizes were sufficient to reveal the binding of antibodies to each population and determine the geometric MFI values.
Data exclusions	No data were excluded from the analyses.
Replication	Independently repeated experiments were carried out to confirm the fluorescence intensity of fluorescent proteins and the binding activities of antibodies. Similar levels of fluorescence intensity or binding activities were detected in repeated experiments.
Randomization	Samples were not randomized as it was not deemed necessary with the experimental setup.
Blinding	Different reporter cell lines were pooled for staining and flow cytometry analysis. The identities of individual cell lines were blinded during the assay. The binding activities of the antibody to individual cell lines were revealed until data analysis.

Reporting for specific materials, systems and methods

We require information from authors about some types of materials, experimental systems and methods used in many studies. Here, indicate whether each material, system or method listed is relevant to your study. If you are not sure if a list item applies to your research, read the appropriate section before selecting a response.

Materials & experimental systems

n/a	Involved in the study	
☐	☒	Antibodies
☐	☒	Eukaryotic cell lines
☒	☐	Palaeontology and archaeology
☐	☒	Animals and other organisms
☐	☒	Human research participants
☒	☐	Clinical data
☒	☐	Dual use research of concern

Methods

n/a	Involved in the study	
☒	☐	ChIP-seq
☐	☒	Flow cytometry
☒	☐	MRI-based neuroimaging

Antibodies

Antibodies used	Commercially available antibodies: hCD32A-FITC (clone IV.3, STEMCELL 60012FI, Lot# 17L84534), hCD32-APC (clone FLI8.26, BD 559769, Lot# 7216899), hCD16-PE (clone 3G8, BioLegend 302007, Lot# B238509), hCD32-PE (clone FLI8.26, BD 550586, Lot# 7349979), hCD64-PE (clone 10.1, BioLegend 305007, Lot# B241142), hCD4 (clone SK3, BioLegend 344602, Lot# B231529), hCD8a (clone HIT8a, BioLegend 300902, Lot# B273962), hCD86 (clone BU63, BioLegend 374202, Lot# B254472), hCD154 (clone 24-31, BioLegend 310802, Lot# B185875), mCD8a-PerCP-eFluor710 (clone 53-6.7, ThermoFisher 46-0081-82, Lot# 4329729), mCD86-PE-Vio770 (clone PO3.3, Miltenyi Biotec 130-105-135, Lot# 5190524011), mCD4-APC-Fire750 (clone RM4-5, BioLegend 100568, Lot# B267119), hCD58-PE (clone MEM-63, ThermoFisher MA1-10256, Lot# TC2530786), HA tag (clone 16B12, BioLegend 901533, Lot# B291379), hCCR2 (clone K036C2, BioLegend 357201, Lot# B255360), hCCR2 (clone 48607, R&D Systems MAB150, Lot# AOT0320031), hCCR5 (clone 2D7, BD 555991, Lot# 9030905), hCCR5 (clone CTC8, R&D Systems MAB1801, Lot# CRV032011A), hCCR5 (clone 45523, R&D Systems MAB181, Lot# AJB1320041), hCCR5 (clone 45529, R&D Systems MAB184, Lot# AWM022011A), hCCR5 (clone 45549, R&D Systems MAB183, Lot# AWL042011B), Mouse IgG1 kappa isotype control (Rockland 010-001-330, Lot# 24773), Goat Anti-Human IgG-PE (Southern Biotech 2040-09, Lot# J4118-VL69B), Goat Anti-Mouse IgG, Human ads-PE (Southern Biotech 1030-09, Lot# L3919-XC40), Human IgG1 kappa (Southern Biotech 0151K-01, Lot# F2317-W020), Human IgG1 lambda (Southern Biotech 0151L-01, Lot# C1615-TC70).<br>Homemade antibodies: FI6, S5V2-29, CH67 and HC19 (all in human IgG1 isotype) were prepared as described in Methods.
Validation	All commercially available antibodies were confirmed for their species and application based on the statements on the manufacturers' websites. Homemade antibodies were validated with recombinant hemagglutinin antigen conjugated beads in Luminex assays.

Eukaryotic cell lines

Policy information about cell lines

Cell line source(s)	HEK 293T and K562 cell lines were purchased from ATCC.
Authentication	None of the cell lines used were authenticated.
Mycoplasma contamination	HEK 293T and K562 cell lines were tested negative for mycoplasma contamination. K562 derivative cell lines were not tested for mycoplasma contamination.
Commonly misidentified lines (See ICLAC register)	No commonly misidentified cell lines were used.

Animals and other organisms

Policy information about studies involving animals; ARRIVE guidelines recommended for reporting animal research

Laboratory animals	Species, Mus musculus; stain, C57BL/6; sex, female; age 12-week-old.
Wild animals	The study did not involve wild animals.
Field-collected samples	The study did not involve samples collected from the field.
Ethics oversight	All experiments involving animals were approved by the Duke University Institutional Animal Care and Use Committee.

Note that full information on the approval of the study protocol must also be provided in the manuscript.

Human research participants

Policy information about studies involving human research participants

Population characteristics	One healthy 37-year-old male participant was recruited.
Recruitment	One healthy participant was recruited to collect PBMC samples, from which CD4, CD8a, CD86, CD154, CCR2b and CCR5 coding sequences were cloned.
Ethics oversight	Duke Institutional Review Board Committee (Pro00062495)

Note that full information on the approval of the study protocol must also be provided in the manuscript.

Flow Cytometry

Plots

Confirm that:

☒ The axis labels state the marker and fluorochrome used (e.g. CD4-FITC).

☒ The axis scales are clearly visible. Include numbers along axes only for bottom left plot of group (a 'group' is an analysis of identical markers).

☒ All plots are contour plots with outliers or pseudocolor plots.

☒ A numerical value for number of cells or percentage (with statistics) is provided.

Methodology

Sample preparation	Cultures of K562 and derivative cells were harvested, centrifuged at 300× g for 2 min at 4°C and resuspended in staining buffer (PBS supplemented with 2% heat-inactivated FBS). After incubation with antibodies at 4°C in the dark for 30 min, cells were washed with staining buffer and resuspended in staining buffer for either secondary staining following the same procedure above or being stored on ice for flow cytometry analysis or single-cell sorting.
Instrument	Flow cytometry analysis was carried out using either BD FACSCanto II cytometer (Duke Cancer Institute Flow Cytometry Shared Resource) or BD LSR II cytometer (The Duke Human Vaccine Institute (DHVI) Research Flow Cytometry Facility). Single-cell sorting was performed with BD Aria II (The DHVI Research Flow Cytometry Facility).
Software	Flow cytometry data were collected with BD FACSDiva (Version 9.0) and analyzed using FlowJo (Version 10.7.2).
Cell population abundance	Post sort clonal reporter cell lines expressing corresponding FP(s) and/or antigen had >95% FP+ and/or antigen+ cells at each stage.
Gating strategy	All live K562 and derivative cells were gated for analysis. For FP channels, two well-separated populations were gated as negatives and positives, respectively. For the detection of binding to analyte antigens, histograms with MFI values above 2-

fold of background (the average MFI value of internal control cell lines K530-FP0000 and K530-FP1111) were scored as positive.

☒ Tick this box to confirm that a figure exemplifying the gating strategy is provided in the Supplementary Information.
